# Life cycle assessment of asphalt pavement containing MSWI bottom ash

**DOI:** 10.1038/s41598-025-97241-7

**Published:** 2025-04-11

**Authors:** Yu Sun, Yucheng Zhong, Jian Liao, Yan Gao, Chongwei Huang

**Affiliations:** 1 University of Shanghai for Science and Technology, Shanghai, China; 2 Shanghai Pudong Road and Bridge Group Co., Ltd, Shanghai, China; 3 Shanghai Lucheng Intelligent Technology Development Co., Ltd, Shanghai, China

**Keywords:** Road engineering, Municipal solid waste incineration bottom ash, Asphalt mixtures, Life cycle assessment, Civil engineering, Sustainability

## Abstract

This paper employs the life cycle assessment method to evaluate the energy consumption and environmental emissions associated with the use of municipal solid waste incinerator (MSWI) bottom ash aggregate (BAA) in asphalt pavement. The analysis divides the life cycle into four stages: the raw material production stage, the pavement construction stage, the operation maintenance stage, and the structural demolition stage. Energy consumption is calculated using the quota method, while environmental emissions are calculated using an emission factor method. Based on an analysis of life cycle inventory data, asphalt pavement containing MSWI BAA shows 9.9% higher energy consumption than conventional asphalt pavement, while both have similar gas emissions throughout their life cycles. Considering the significant consumption of domestic waste incineration bottom ash in asphalt pavement containing MSWI BAA, these advantages are believed to outweigh the disadvantages. This research provides relevant data for the application of bottom ash in asphalt pavement engineering, promoting its development and implementation.

## Introduction

In the context of waste classification, incorporating municipal solid waste incinerator (MSWI) bottom ash in road engineering not only helps conserve natural aggregates but also addresses the issue of reusing bottom ash resources. MSWI bottom ash refers to the solid product remaining after the incineration of domestic waste and consists of sintered bottom ash, glass, metals, and unburned material, among others, making it a form of lightweight aggregate.

The bottom ash aggregate (BAA) can be blended with cement gravel or asphalt mixtures, with Marshall test parameters meeting specification requirements^1^. Replacing natural aggregate with a 20% mass fraction of BAA in AC-20 and 5–10% mass fraction in SMA-13 reduces the adverse effects of aging on low-temperature crack resistance while increasing the fatigue life of the asphalt mixture^2^. Furthermore, freeze‒thaw cycle splitting strength tests demonstrate that the characteristics of bottom ash contribute to an increased internal friction angle of the mixture, resulting in higher structural strength for the asphalt mixture containing MSWI BAA^1^.

Numerous studies have been conducted globally on the feasibility of incorporating bottom ash into road construction. Xu and Alae examined the thermal characteristics and temperature distribution of flexible pavements containing MSWIRs with hollow microsphere structures^3^. Xu and Du explored the rheological characteristics and fatigue performance of asphalt mastics and mixtures containing MSWI residue^4^. The performance of the subgrade and various unbound and bound pavement structural layers are determined for Audrius Vaitkus with bottom ash, aiming to develop an algorithm for the use of MSWI bottom ash as a building material^5^. Tang and Zhang utilized cement and a chelating agent to solidify fly ash and immobilize polluting elements. The potential for using solidified fly ash in pavement materials has also been assessed from mechanical and environmental perspectives^6^. Jinwei Yao investigated the effect of replacing natural sand with MSWI bottom ash on the macro- and microscopic performance of cement- based materials after wet treatment^7^. Through finite element method, Ying Yuan conducted a numerical simulation study on the influence of the interfacial zone one the tensile damage behavior of asphalt mixture contained BAA under indirect tensile force^8^. Kyungwon Park researched the positive effect of the combined use of recycled asphalt shingles and municipal solid waste incineration bottom ash in asphalt concrete^9^. Yao Zhao’s experimental result shows that the leachate from the MSWI–BAA and PAC–13 mixture with MSWI – BAA was shown to be safe for irrigation^10^. Lynn analyzed the environmental impacts of MSWI bottom ash as a potential construction material in road pavements and geotechnical applications^11^.

To achieve the goal of low-carbon environmental protection, it is necessary to conduct a quantitative analysis of the energy consumption and environmental emissions associated with asphalt pavement containing MSWI BAA before its widespread adoption. The widely used method for analyzing the energy consumption and environmental emissions of projects is the life cycle assessment method (LCA). Oreto and Biancardo evaluated sustainability criteria during the design stage using both life cycle assessment and life cycle cost analysis methods^12^. Chen and Wang quantified greenhouse gas emissions from asphalt pavements containing reclaimed asphalt pavement using LCA from a temporal perspective^13^. Xinxing Zhou selected one representative cement concrete pavement and two asphalt mixtures highways for quantitative evaluation and LCA analysis^14^. Zhang and Wang used LCA to assess the energy consumption and emission characteristics of asphalt pavement with different design life spans^15^. Yang performed LCA analysis on the production, mixing, spreading, and rolling process of traditional mix and modified warm mix^16^. Zheng developed a comprehensive pavement LCSA methodology that integrated life-cycle cost analysis, environmental life-cycle assessment, and social life-cycle assessment^17^. Shi applied the economic input-output life cycle assessment approach to assess the life cycle of RCA-based Portland cement concrete pavements and ordinary PCC pavements from all three aspects of sustainability namely economic impact, social impact, and environmental impact^18^. Buttitta proposed an assessment framework consisting of a life cycle assessment and a life cycle cost assessment to calculate the carbon footprint of pavement materials and pavement activities and to determine the overall economic burden and maintenance strategy of the relevant pavement over the 60-year analysis period^19^. In the case of increasing maintenance demand and limited maintenance budget, Huang introduced a maintenance decision method that integrates LCA, LCCA and multi-objective optimization, and comprehensively considers three dimensions of performance, economy, and environment to establish a maintenance decision system^20^.

In summary, research on asphalt pavement containing MSWI BAA is still in its early stages globally, and there is a lack of research on energy consumption and environmental emissions throughout the life cycle of such pavements. This paper focuses on the surface layer of asphalt pavement containing MSWI BAA as the research object. The LCA is employed to calculate and analyze the energy consumption and environmental emissions at each stage: raw material production, pavement construction, operation and maintenance, and structural demolition. The findings provide guidance for using BAA in asphalt pavement.

## Life-cycle assessment

### Definition of life-cycle assessment

The Life-Cycle Assessment (LCA) method involves the compilation and assessment of the inputs, outputs, and potential environmental impacts throughout the entire life cycle of a product system. By using the LCA method, a product or process can be systematically evaluated, encompassing raw material acquisition, product production, use, and post-use disposal. LCA enables the evaluation of all environmental impact factors that can be considered, including resource utilization, energy consumption, and atmospheric emissions.

### Technical framework of LCA

**Fig. 1:**
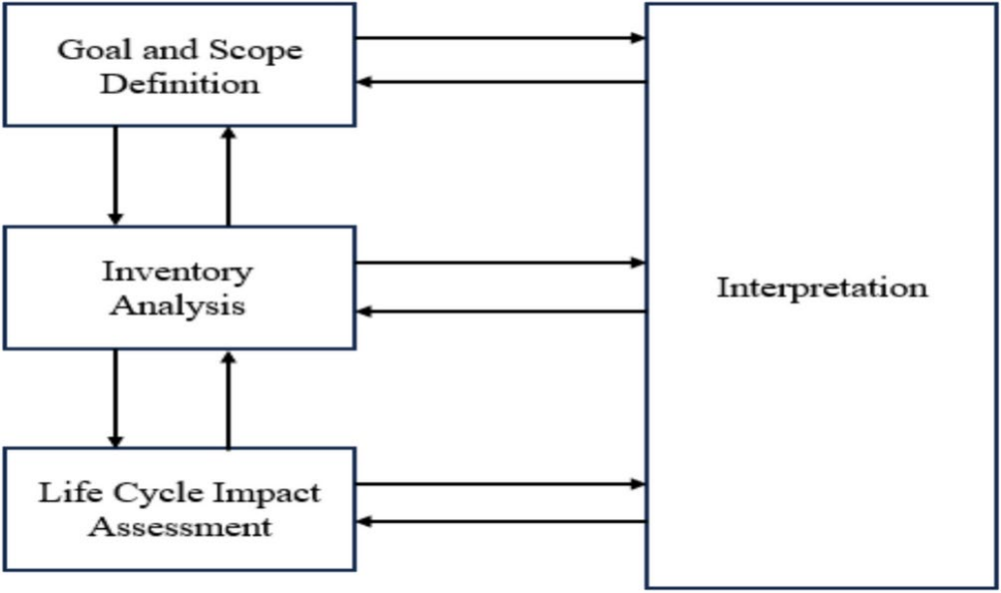
LCA framework.

According to the technical framework defined by ISO14040^21^, the LCA evaluation process includes four parts: goal and scope definition, inventory analysis, impact assessment, and interpretation, as shown in Fig. 1.

### Life cycle assessment steps for asphalt pavement containing MSWI BAA

The life cycle assessment steps for asphalt pavement containing MSWI BAA are as follows: ① clarify the product system, focusing on the surface layer of asphalt pavement containing MSWI BAA and using ordinary asphalt pavement as the control group; ② determine the raw material consumption; ③ establish the system boundary, dividing the whole life cycle of asphalt pavement containing MSWI BAA into four stages: raw material production, pavement construction, operation and maintenance, and structural demolition, with the research boundary selecting the raw material production stage;④ determine the LCA energy consumption and emission model and calculation method for the road; ⑤ conduct LCA inventory analysis; and ⑥ interpret the LCA inventory results.

## Life cycle study of asphalt pavement containing MSWI BAA

### Goal and scope definition

According to the provisions of the national standard Environmental management—Life cycle assessment—Principles and framework (GB/T 24044-2008), the scope of the LCA study on asphalt pavement containing MSWI BAA in this paper specifically includes the asphalt pavement containing MSWI BAA product system, system boundaries, and functional units, and establishes a systematic model.

#### Product system

**Fig. 2:**
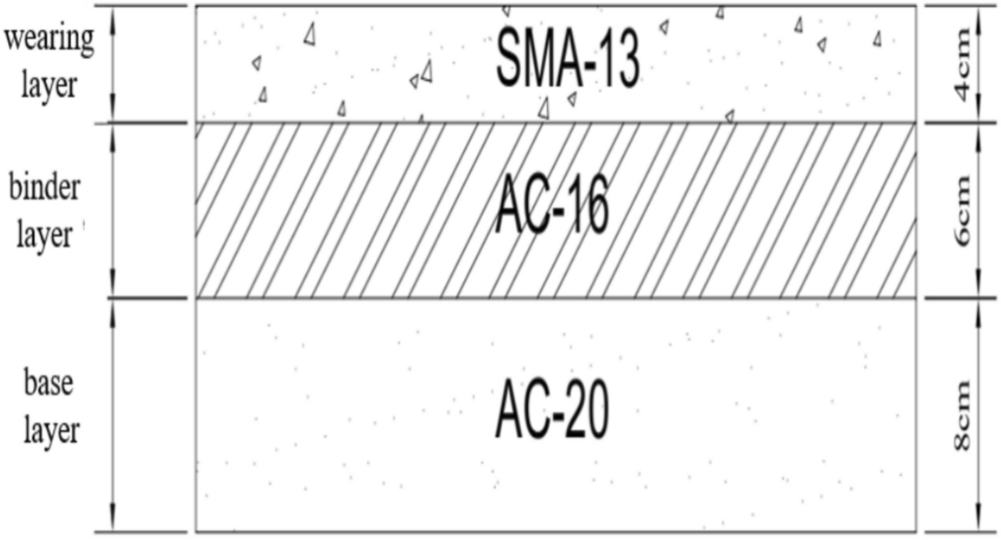
Asphalt pavement surface layer structure diagram.

The study’s subject is asphalt pavement containing MSWI BAA, with conventional asphalt pavement serving as the control group. According to the *Technical Standard of Highway Engineering (JTB01-2014)*, the pavement structure’s design standard axle load is a dual-wheel single axle of 100 kN, with a tire pressure of 0.7 MPa. The road under study is a two-way four-lane first-class highway with a design speed of 80 km/h, a subgrade width of 27 m, and a road surface width of 24 m. These included a 2 × 0.5 m soil shoulder + 2 × 3 m hard shoulder + 4 × 3.75 m lane + 2 m central divider. The design service life of both asphalt pavements is 15 years, and related research has focused on asphalt pavement surfaces. The wearing, binder, and base layers of the asphalt pavement consist of SMA-13, AC-16, and AC-20 asphalt mixtures, respectively, with thicknesses of 4, 6, and 8 cm. The particle size range and content of BAA used in this study are as follows: the particle size range of BAA mixed with the SMA-13 BAA asphalt mixture is 0–2.36 mm, with a content of 10% (by mass fraction of mineral aggregate); the particle size range of BAA in the AC-16 and AC-20 BAA asphalt mixture is 0–9.5 mm, with a content of 20%. As shown in Fig. 2.

#### System boundary

The environmental impact analysis of the pavement using the LCA method requires a reasonable division of the entire life cycle process of the pavement. In this study, the entire life cycle process of the pavement is divided into four main stages based on the research objectives and content:

① Raw Material Production and Processing Stage. This stage involves the extraction and processing of aggregates, treatment and screening of bottom ash, refining of crude oil, production of asphalt, and the transportation of materials from the production site to the construction site; ② This stage includes the mixing, transportation, paving, and compaction of asphalt pavement materials. It covers processes such as the heating and mixing of raw materials, as well as the energy consumption and emissions associated with machinery used during the transportation, paving, and compaction phases; ③ This stage encompasses all activities that take place after the pavement is constructed. It considers the environmental impacts of the pavement, including factors such as surface reflection, vehicle rolling resistance, and pavement carbonation; ④ Pavement Structural Demolition Stage. This stage involves maintenance, recycling, and reconstruction activities that occur during the pavement’s life cycle.

**Fig. 3:**
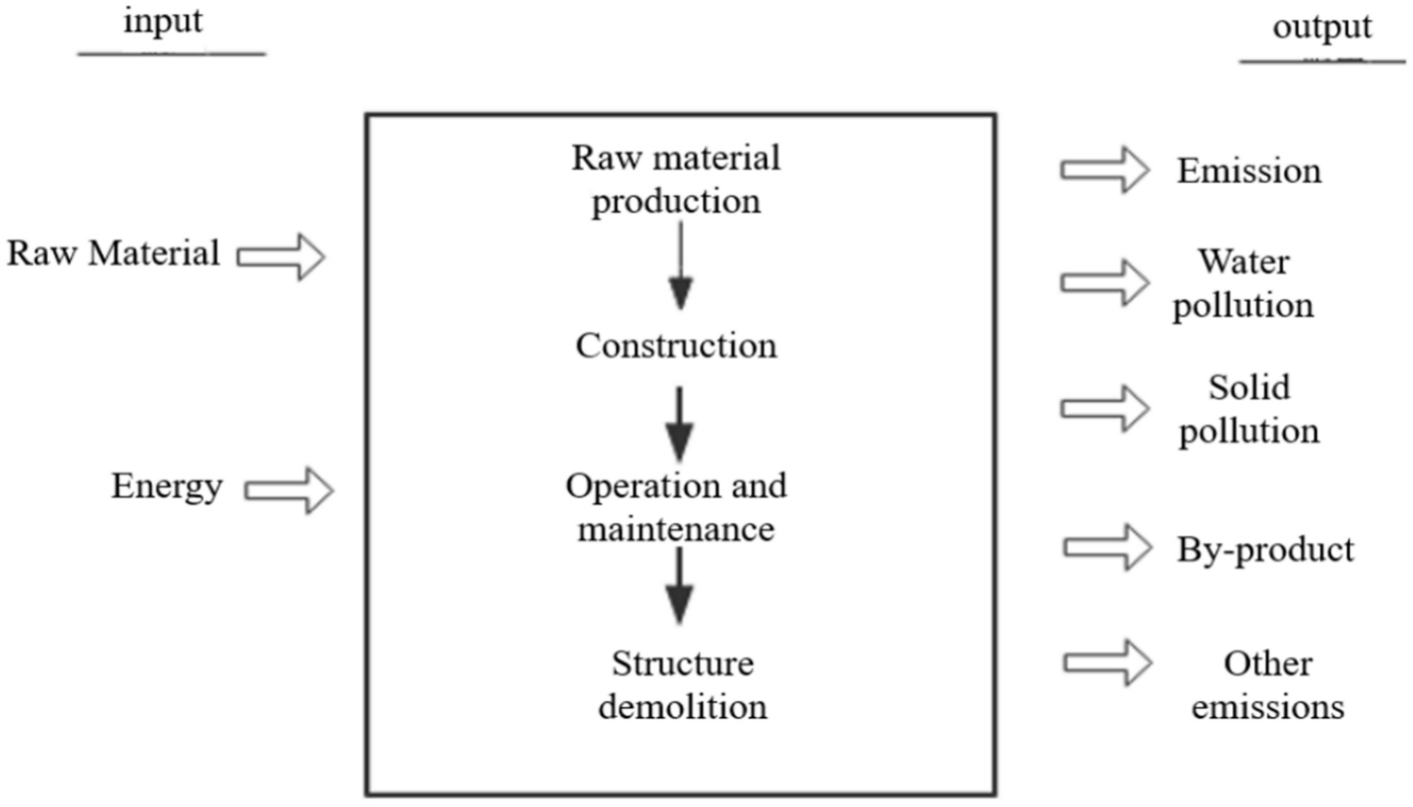
System boundary.

The system boundary for this study is illustrated in Fig. 3, with inputs consisting of raw materials and energy, and outputs representing the emissions of various wastes.

#### Functional unit

For this research, a 1 km two-way four-lane highway is selected as the functional unit. The input represents the energy consumed throughout the life cycle of the 1 km highway, measured in MJ/functional units. The output refers to the emissions generated during the life cycle of 1 km of highway pavement, measured in g/functional unit.

#### Systematic model

**Fig. 4:**
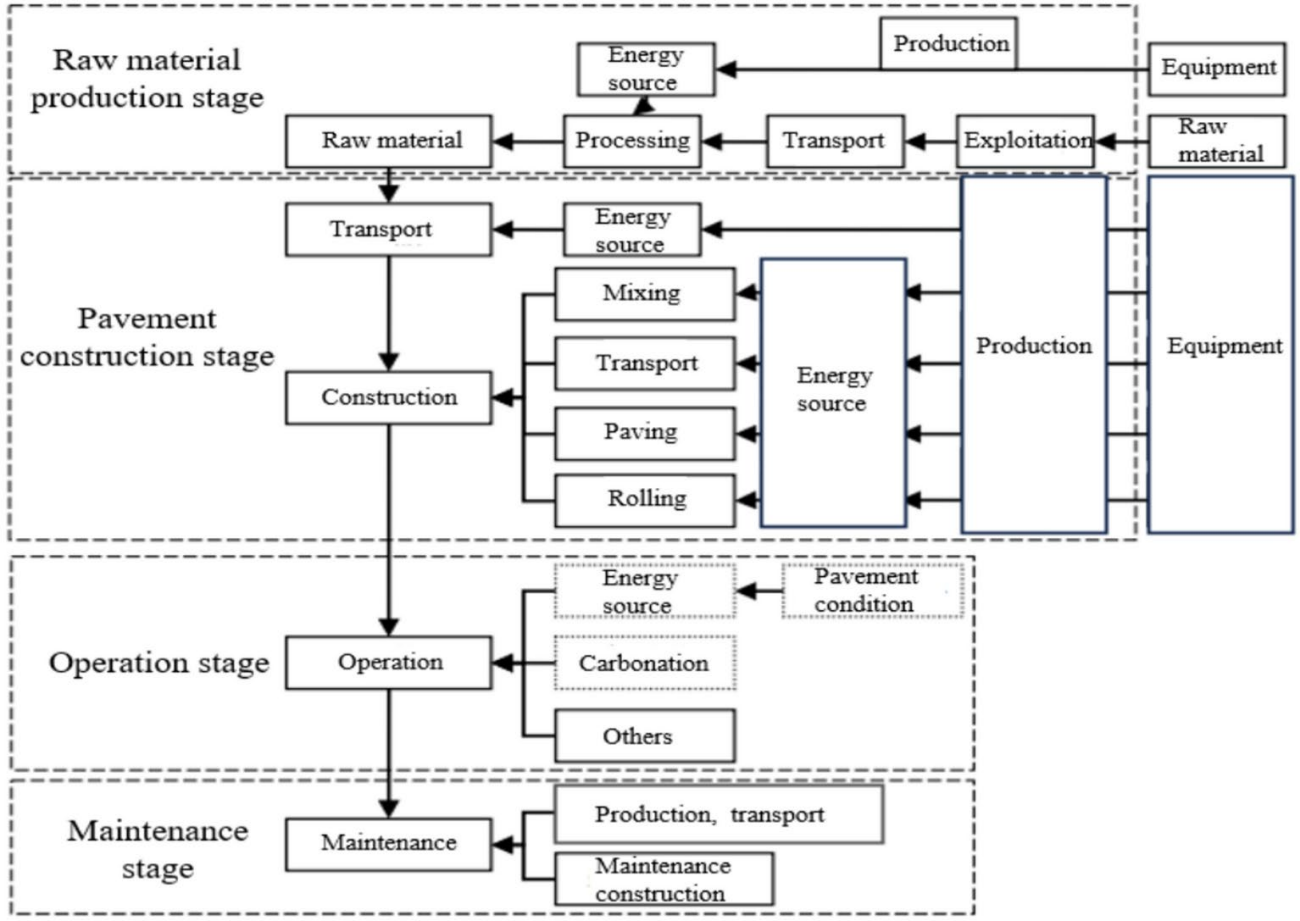
Systematic model.

According to the system boundary and the four stages of pavement life cycle, the life cycle analysis model of pavement system is established, as shown in Fig. 4.

### Life cycle inventory analysis of pavement

The analysis of energy consumption and emissions during the operation and maintenance stage of the pavement life cycle is quantitatively performed. The inventory analysis focuses on collecting relevant data on energy consumption and emissions and selecting the appropriate calculation method for quantification. The primary environmental impact factors at each stage are presented in Table 1.

**Table 1:** Primary environmental impact factors at each stage of the pavement life cycle.

Life cycle stage	Environmental factors	Indicators
Raw material production stage	The production, processing, and transportation of raw materials	Energy consumption and emissions in the asphalt production stage, the stone production stage and the BAA production stage
Pavement construction stage	The mixing, transportation of asphalt mixture and the operation of mechanical equipment such as pavement paving and compaction	Energy consumption and emission from asphalt mixture mixing Energy consumption and emissions of asphalt pavement construction process
Operation maintenance stage	Increased vehicle fuel consumption due to changes in road conditions during operation, operation of raw materials and construction machinery during road maintenance	Energy consumption based on the rutting damage Environmental emission based on rutting damage maintenance
Structure demolition stage	Construction machinery for pavement demolition structure operation	

#### Method for calculating pavement life cycle energy consumption and emission data

The quota method is utilized in this study to calculate energy consumption. By multiplying the number of mechanical shifts required in the construction of physical pavement per unit volume or area by the energy consumption parameters of each shift, the energy consumption required to produce physical pavement per unit volume or area can be determined. The energy consumption data obtained using this method are expressed in terms of the mass or volume of various fuels, such as diesel, gasoline, and heavy oil. These data are then uniformly converted into MJ based on the heating value of the fuels.

IPCC mainly focuses on clean fuels, and due to the lack of heavy oil refining processes, there is less support in the life cycle database, and there are fewer emission factors for specific application scenarios, so the emission factors of heavy oil are not considered here.

This paper summarizes the net heating value and gas emission factors of commonly used fuels and electricity in asphalt pavement engineering, as shown in Table 2. Both the fuel emission factor and the electrical energy emission factor are sourced from *2019 Refinement to the 2006 IPCC Guidelines for National Greenhouse Gas Inventory*^22^.

**Table 2:** Net calorific value and emission factors of commonly used fuels and electricity.

Energy types	Energy consumption/MJ	Fuel emission factor/g		
		CO_2_	CH_4_	N_2_O
Automotive gasoline (1 kg)	44.3	2990	0.129	0.026
Gasoline/Diesel blend (1 kg)	43.0	3160	0.128	0.026
Heavy oil (1 kg)	40.4	–	–	–
Electricity (1kWh)	3.6	829	0.01169	0.01692

Energy consumption is determined using the quota method, while environmental emissions are calculated through the emission factor method, as depicted in Eq. (2.1).

2.1 $$E = \alpha \times EF \times \left( {1 - \frac{ER}{{100}}} \right)$$

where $E$ is the emission quantity, kg; $\alpha$ is the activity quantity, kg; $EF$ is the emission factor, g• kg^-1^, and $ER$ is the reduction efficiency.

#### Impact assessment and result interpretation

The purpose of Life Cycle Impact Assessment (LCIA) is to conduct a qualitative and quantitative analysis of the environmental impact factors identified in the inventory analysis, in order to determine the extent of impact that material and energy exchanges during the product life cycle have on the external environment. The interpretation of results involves combining the findings from inventory analysis and impact assessment to draw conclusions and provide recommendations.

##### Results categorization

This paper focuses on the gas emissions as the studied emission type, with greenhouse gases, acidifying agents, and gases harmful to human health as the main research categories. Categorizing the inventory analysis results involves grouping emissions with similar impacts, exploring the intensity of influencing factors, determining the percentage of pollutant emissions, and determining their range. For instance, gases such as SO_2_ and NO_x_, which contribute to acidification, should be grouped together for analysis.

##### Characterization of the classification results

The proportion of emissions varies for different environmental impact factors within the same impact category. Therefore, it is necessary to transform the data using characterization factors before aggregation. The characterization formula is presented in Eq. (2.2):

2.2 $$E_{i} = \sum\limits_{j} {(I_{ij} \times P_{{{\text{ij}}}} )}$$

where *E*_*i*_ is the characterization result for the *i-th* impact category, kg; *I*_*ij*_ is the inventory analysis result for the *j-th* impact factor in the *i-th* impact category, kg; and *P*_*ij*_ is the characterization factor for the *j-th* impact factor in the *i-th* impact category.

In this study, the characterization of the categorized results from the life cycle energy-saving and emission reduction inventory analysis of asphalt pavements is conducted using an equivalent model, as shown in Eq. (2.2). The impact categories, impact factors, and corresponding characterization factors for each impact factor are presented in Table 3 ref.^23^.

**Table 3:** Impact categories, impact factors and characteristic factors.

Impact categories		Impact factors	Characteristic factor unit	Characterization factor
Material consumption		Aggregates, asphalt	t	1
Energy consumption		Gasoline, diesel, liquefied petroleum gas	MJ	1
Environmental emissions	Greenhouse gas	CO_2_	kg CO_2_ equivalent (IPCC 100-year model)	1
		CH_4_		25
		N_2_O		298
	Acidifying gas	SO_X_	kg SO_2_ equivalent	1
		NO_X_		0.7
		NH_3_		1.88
	Harmful gas	CO	kg 1,4-dichlorobenzene equivalent	2.4
		NMVOC		0.64
	Particulate matter	PM_10_、PM_2.5_	kg	1

## Life cycle inventory data of asphalt pavement containing MSWI BAA

### Raw materials

#### Natural aggregates and fillers

**Fig. 5:**
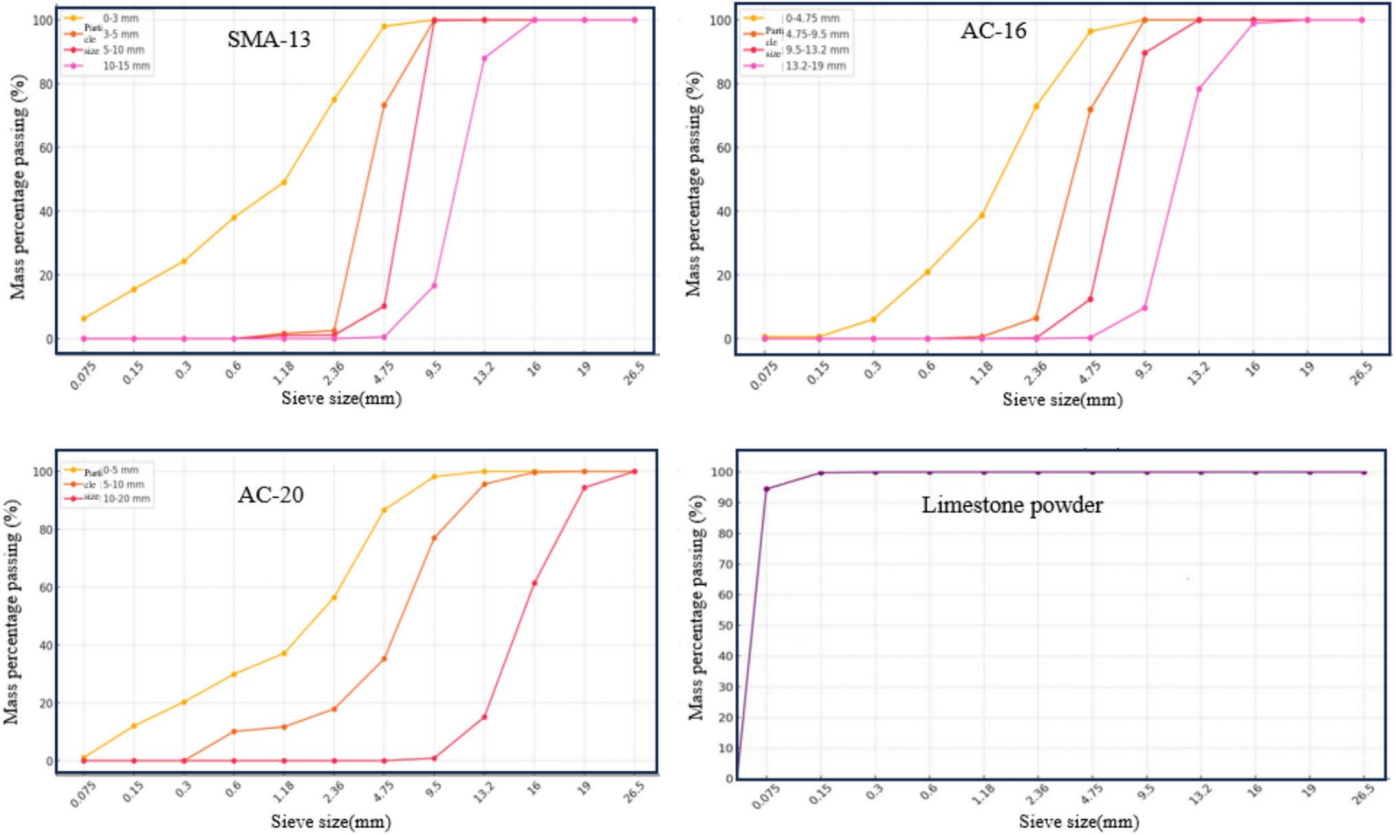
Sieving results for each aggregate grade.

The composition design and performance testing of the AC-20 mixture uses three-grade limestone aggregate, while the AC-16 and SMA-13 mixtures use four-grade basalt aggregate for their composition design and performance testing. The filler used in the composition design and performance testing of the AC-20, AC-16, SMA-13, and the other three mixtures was natural limestone mineral powder. The particle size range and main performance indicators of each grade of aggregate are shown in Table 4, and the sieving results are presented in Fig. 5.

**Table 4:** The main performance indicators of each grade of aggregate.

Mixture type	Aggregate type	Particle size range/mm	Apparent relative density	Bulk volume relative density	Water absorption rate/%
SMA-13	Basalt	0–3	2.863	–	–
		3–5	3.009	2.806	2.41
		5–10	2.998	2.815	2.17
		10–15	2.985	2.841	1.70
AC-16	Basalt	0–4.75	2.911	–	–
		4.75–9.5	2.996	2.776	2.65
		9.5–13.2	2.985	2.776	2.52
		13.2–19	2.986	2.823	1.93
AC-20	Limestone	0–5	2.708	–	–
		5–10	2.825	2.793	0.43
		10–20	2.739	2.712	0.36
Limestone powder		0–0.075	2.667	–	–

#### BAA

##### Appearance

**Fig. 6:**
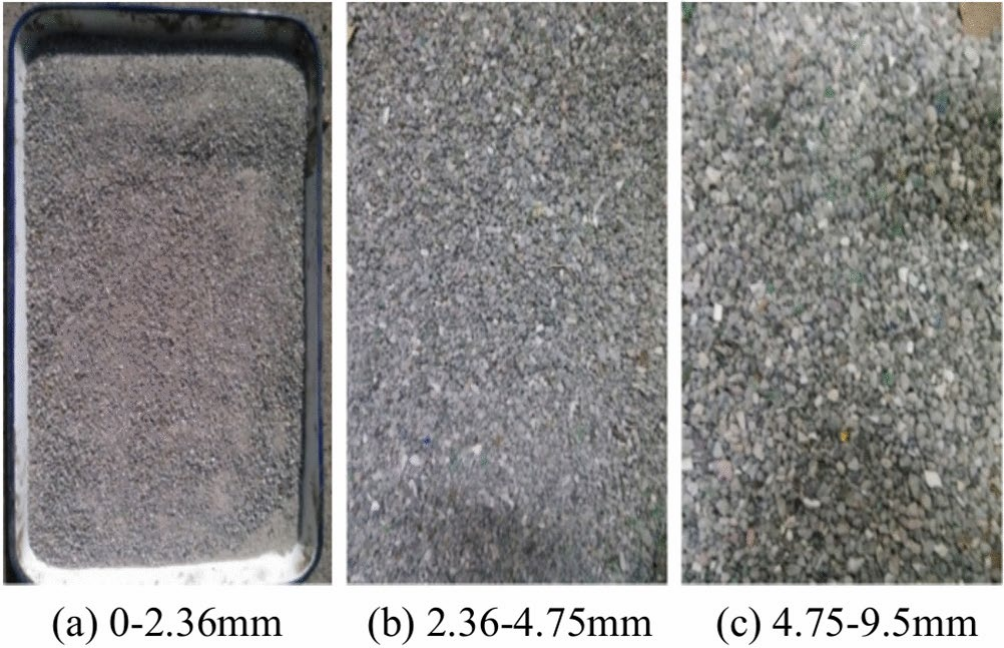
The appearance characteristics of BAA with different particle size ranges.

The appearance of BAA (0–2.36mm, 2.36 -4.75mm, 4.75–9.5mm) obtained by sieving BAA with a particle size range of 0–9.5mm is shown in Fig. 6. The BAA with particle size ranges of 2.36–4.75 mm and 4.75–9.5 mm exhibit varied colors and contain more impurities, showing a clean difference from natural aggregates. This is due to the presence of metals, glass, ceramics, bricks and stones in the BAA. In contrast, the BAA with a particle size of 0–2.36 mm have a relatively uniform color and are similar in appearance to natural aggregates.

##### Material composition

**Fig. 7:**
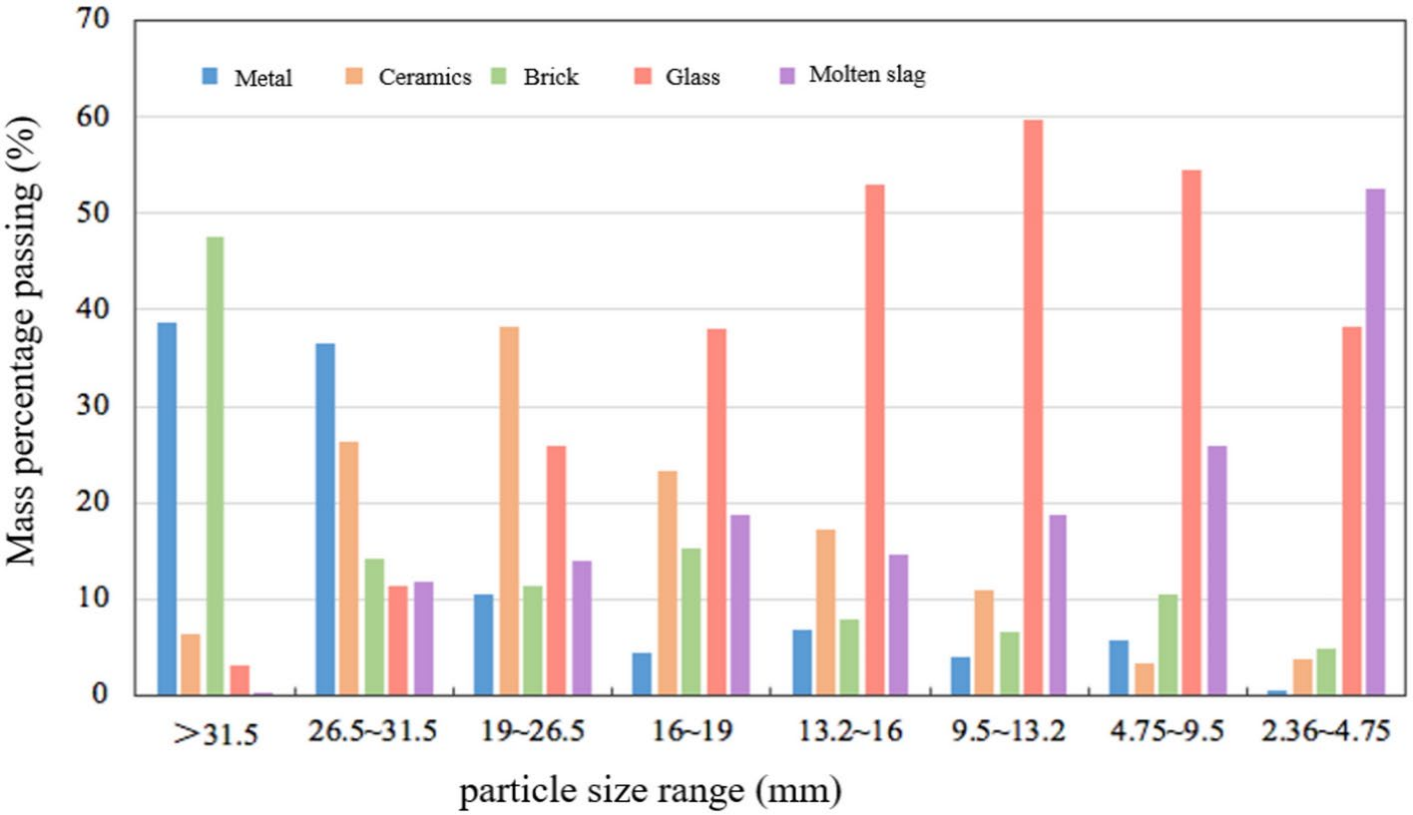
Material composition distribution of BAA. The BAA in the particle size range of 0–2.36 mm were too small to sort, so the material composition of this portion is not shown in the figure.

In order to clarify the material composition of the BAA, manual sorting of the BAA was conducted to determine the distribution of different components, and the results were presented in Fig. 7.

Figure 7 reveals that BAA particles larger than 2.36 mm are primarily composed of metals, ceramics, bricks, glass, and molten ash with only molten ash being a byproduct of the waste incineration process. Metals, ceramics, glass, and bricks originate from the raw composition of municipal waste. In the BAA particles larger than 26.5 mm, solid wastes such as ceramics, bricks, and metals predominate, with less molten ash present. As the particle size decreases, the metal content in BAA sharply decreases, while the contents of glass and molten ash increase, and the levels of ceramics and bricks remain relatively stable. Due to the excessive flaky content in BAA particles larger than 9.5 mm, they are unsuitable for use in asphalt mixtures; therefore, this study focuses on the particle size range of 0–9.5 mm.

##### Mechanical property

**Fig. 8:**
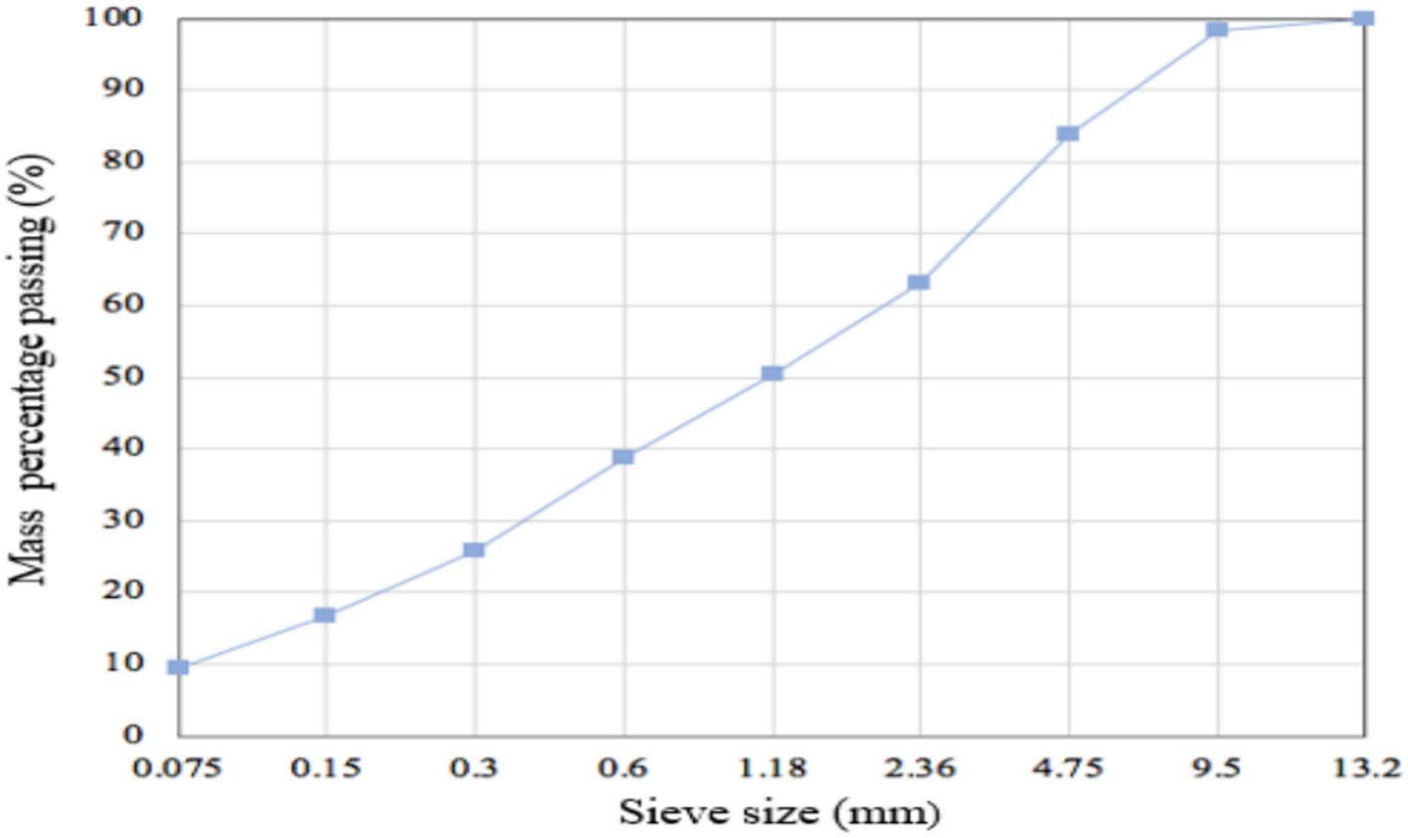
The particle size distribution of BAA.

A sieving test was conducted on BAA particles in the 0–9.5 mm range, and the resulting gradation curve is shown in Fig. 8. As indicated by Fig. 8, the BAA particles are uniformly distributed across particle sizes from large to small, with each size fraction combined in specific proportions. The gradation curve is smooth and continuous, indicating that the BAA belongs to the continuously graded type of aggregate.

**Fig. 9:**
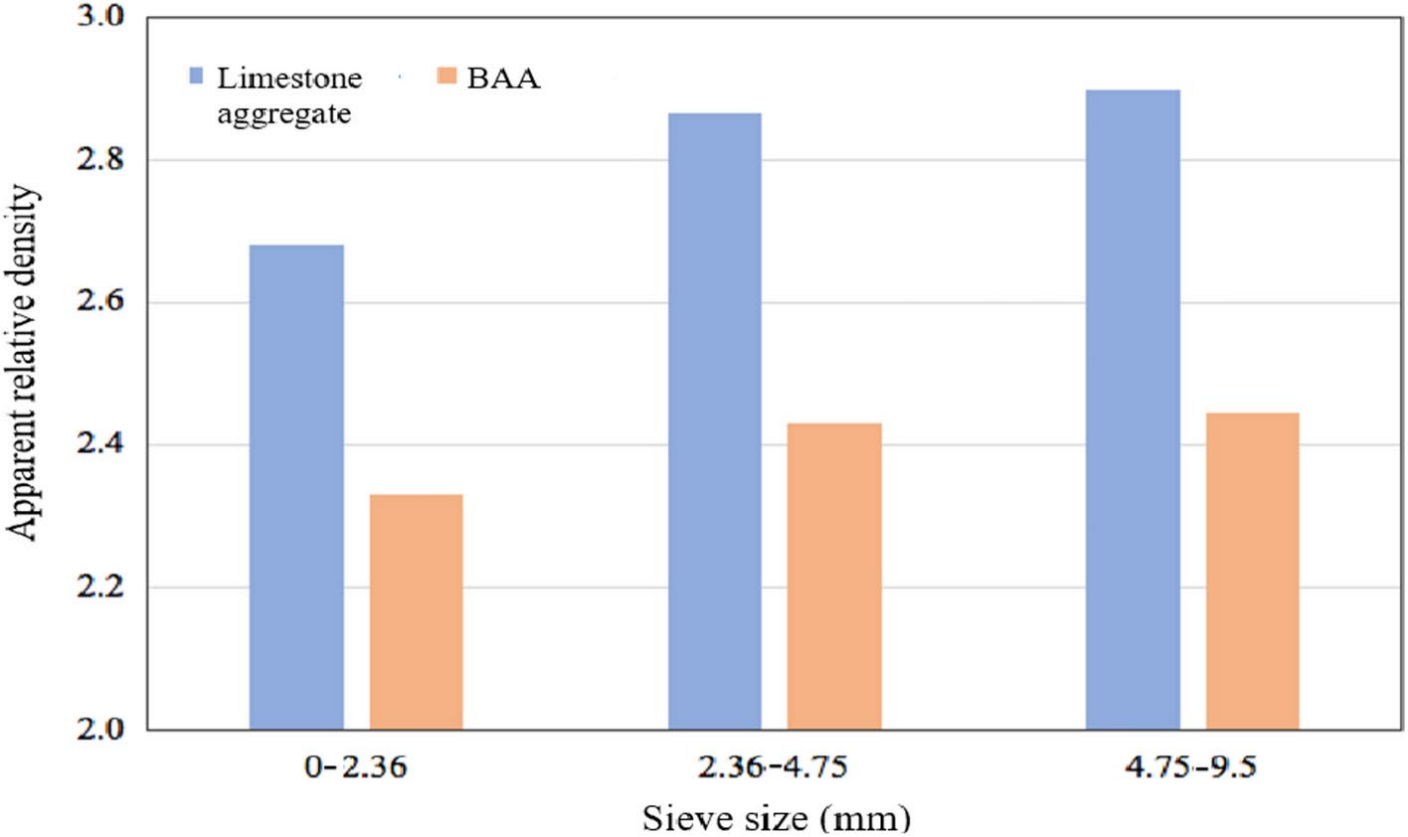
Apparent relative density.

The BAA with a particle size larger than 2.36 mm were tested according to the basket method, while the particles smaller than 2.36 mm were tested according to the volumetric flask method. Limestone aggregate was used as the control group. The test results are shown in Fig. 9.

As shown in Fig. 9, overall, the apparent relative density of BAA is lower than that of natural limestone aggregate across all particle size ranges, approximately 85% of the apparent relative density of limestone. For BAA, the apparent relative density in the 0–2.36 mm range is slightly lower, while the apparent relative densities in the 2.36–4.75 mm and 4.75–9.5 mm ranges are relatively similar.

**Fig. 10:**
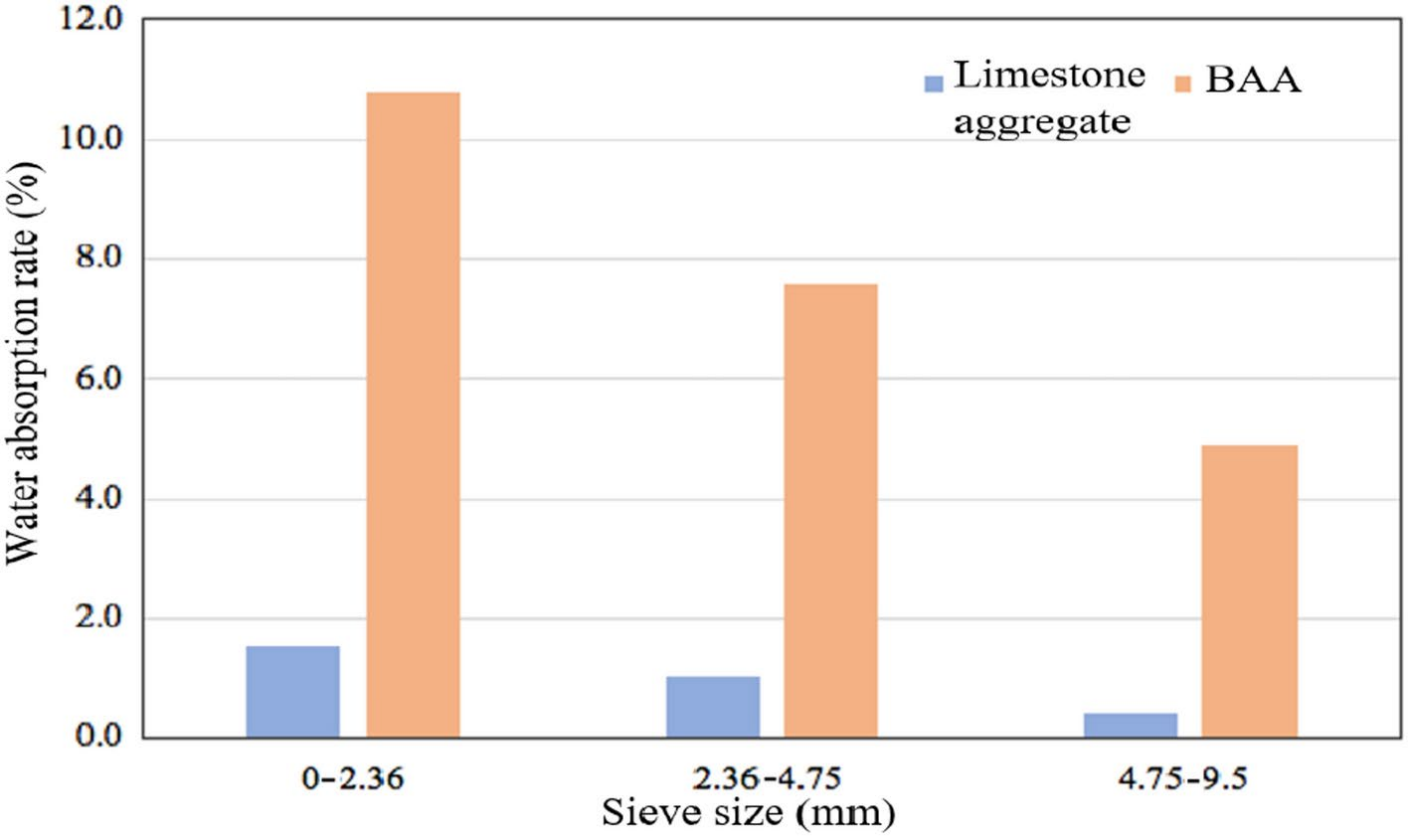
Water absorption rate.

The water absorption tests were conducted separately on BAA and limestone aggregates, and the test results are shown in Fig. 10.

As shown in Fig. 10, overall, the water absorption rate of BAA is significantly higher than that of limestone aggregate across all particle size ranges, approximately 6 to 10 times higher than that of limestone aggregate. This is attributed to the loose, porous structure and rough surface characteristics of BAA particles. The higher water absorption rate of BAA may lead to a higher design asphalt content in BAA-asphalt mixtures. For BAA, as the particle size increases, the water absorption rate gradually decreases, which is related to the material composition of BAA. Larger particle sizes contain a higher proportion of glass and ceramics, which, compared to bricks and slag, have relatively smooth surfaces and less developed porosity, thus leading to a reduction in water absorption.

**Fig. 11:**
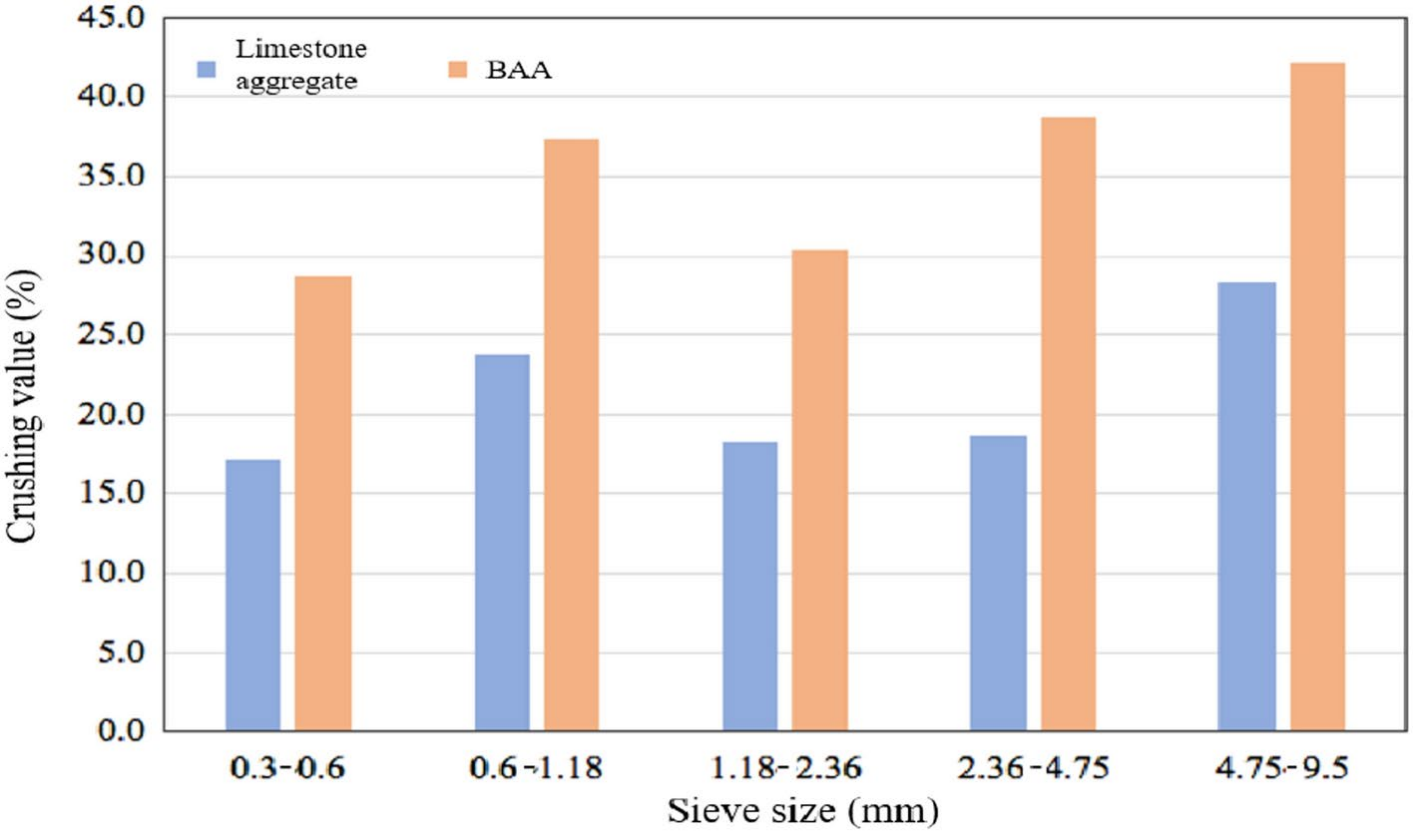
Crushing value.

The crushing value is used to measure the ability of aggregates to resist crushing under gradually increasing loads, serving as a relative indicator of aggregate strength. The crushing values of BAA and limestone aggregate are presented in Fig. 11.

As shown in Fig. 11, overall, the crushing value of BAA is higher than that of natural limestone aggregate across all particle size ranges, approximately 1.6 times that of limestone aggregate. This is related to the loose, porous nature of BAA particles, which are rich in materials such as glass and ceramics. For BAA, the crushing value generally increases with particle size. This is because, in larger particle size ranges, the proportion of glass and ceramics, which have lower strength, increases, thereby reducing the overall mechanical strength of BAA.

##### Chemical composition

**Fig. 12:**
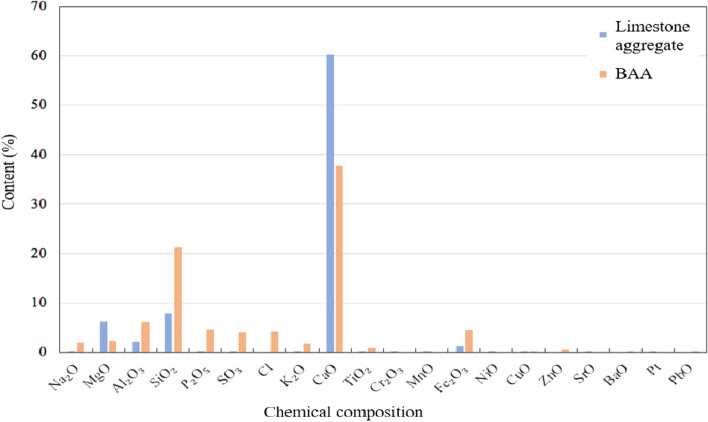
Chemical composition of limestone aggregate and BAA.

After thoroughly grinding and drying both natural limestone and BAA, their chemical compositions were tested using an X-ray fluorescence spectrometer (XRF), model SRS 3400* produced by BRURER AXS in Germany. The test results are shown in Fig. 12. It is important to note that the detectable element range for this test is from O8 to U92, with concentration ranges from ppm to 100%. The test actually measures the elemental content, but the results are presented in the form of oxides corresponding to the element’s valence state.

As shown in Fig. 12, the primary element in limestone is calcium (Ca), which aligns with its main component, CaCO_3_. In addition, small amounts of Si, Mg, Al, and other elements are present. For BAA, the highest content is also Ca, followed by Si, with smaller amounts of Na, Mg, Al, P, S, Fe, and other elements. This shows that the composition of BAA is much more complex than that of natural limestone aggregate.

#### Asphalt

The AC asphalt mixture and SMA asphalt mixture were composed of CNOOC 70# matrix asphalt and I-D type SBS-modified asphalt, respectively, from the SK Company in South Korea. The technical indexes of the asphalt binder were tested, and the test results are shown in Table 5.

**Table 5:** Technical indexes of the asphalt binders.

Asphalt binder	Testing indicator	Testing temperature/℃	Test result	Specification requirement
A-70#	Ductility/cm	10	> 100	≥ 25
	Penetration/0.1 mm	25	72.6	60–80
	Softening point Point/℃	–	47.3	≥ 46
SBS	Ductility/cm	5	78.0	≥ 20
	Penetration/0.1 mm	25	42.1	40–60
	Softening point Point/℃	–	79.1	≥ 60

### Asphalt mixture design proportion

**Fig. 13:**
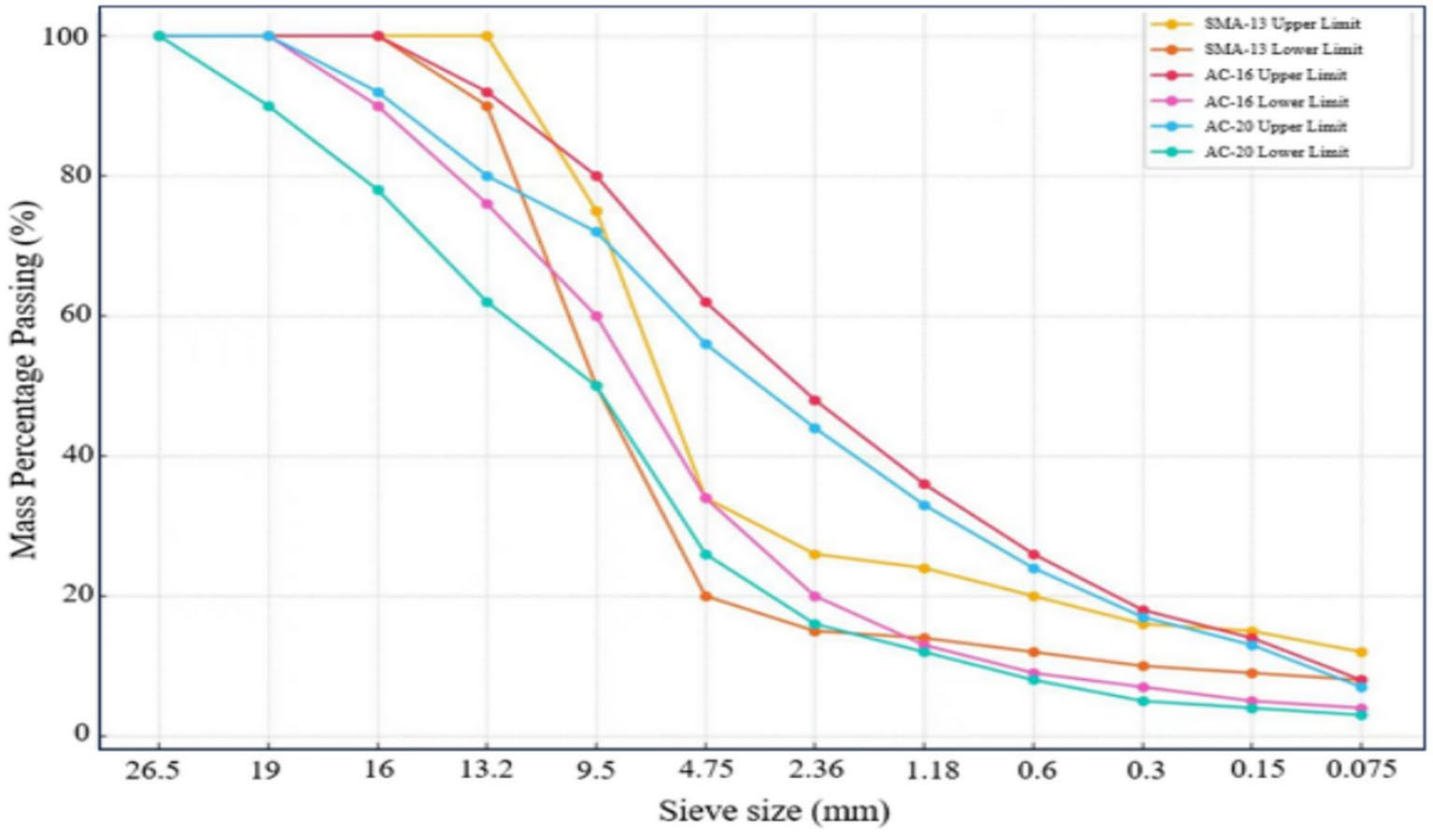
The aggregate grading range for asphalt mixtures.

The Marshall design method is used to designate the mix ratio of the SMA-13, AC-16, and AC-20 asphalt mixtures, in which the aggregate grading range should meet the requirements of Fig. 13.

The design of the mix proportions for the control group asphalt mixture, consisting of the AC-20, AC-16, and SMA-13, was conducted using natural aggregate and natural limestone mineral powder. The code names assigned to these mixtures were A0, A0’, and S0. Subsequently, the BAA was incorporated to replace a portion of the natural aggregate, resulting in mix ratio designs for the BAA asphalt mixtures AC-20, AC-16, and SMA-13, denoted by A1, A1’, and S1, respectively.

The mix designs for the various asphalt mixtures can be found in Table 6. The Marshall test results under the optimum oil-stone ratio are provided in Table 7.

**Table 6:** Mix design results.

Mixture type	Mixture code	BAA content/%	Quantity of natural aggregate in the following particle size ranges (mm)/%				Asphalt-stone ratio/%	Mineral powder/%
			0–5	5–10	10–20	–		
AC-20	A0	0	34	31	33	–	4.1	2
	A1	20	17	28	34	–	5.6	1
Mixture type	Mixture code	BAA content/%	Quantity of natural aggregate in the following particle size ranges (mm)/%				Asphalt-stone ratio/%	Mineral powder/%
			0–4.75	4.75–9.5	9.5–13.2	13.2–19		
AC-16	A0’	0	35	6	13	37	4.2	9
	A1’	20	17	8	10	37	6.1	8
Mixture type	Mixture code	BAA Content/%	Quantity of natural aggregate in the following particle size ranges (mm)/%				Asphalt-stone ratio/%	Mineral powder/%%
			0–3	3–5	5–10	10–15		
SMA-13	S0	0	14	0	30	45	6.5	11
	S1	10	1	5	29	45	7.5	10

**Table 7:** Marshall test results for the experimental asphalt mixtures.

Mixture type	Mixture code	Optimal asphalt-stone ratio/%	VCA_DRC_/%	VCA_mix_/%	Bulk volume relative density	VMA/%	VFA/%	MS/kN	FL/mm
AC-16	A0’	4.2	–	–	2.490	15.7	70.5	8.7	3.6
	A1’	6.1	–	–	2.387	18.4	77.8	11.1	4.0
	Technical requirement		–	–	–	≥ 13.5	65–75	≥ 8	1.5–4
AC-20	A0	4.1	–	–	2.457	13.1	68.0	8.6	3.8
	A1	5.6	–	–	2.354	14.9	74.8	9.4	3.7
	Technical requirement		–	–	–	≥ 13	65–75	≥ 8	1.5–4
SMA-13	S0	6.5	41.5	40.2	2.478	17.7	76.7	10.6	4.2
	S1	7.1	41.5	41.1	2.418	18.3	78.1	12.3	4.7
	Technical requirement		VCA_DRC_ ≥ VCA_mix_		–	≥ 17	75–85	≥ 6	–

### Asphalt pavement quantity

#### Asphalt pavement containing MSWI BAA engineering quantity

The total volume of the mixture required for the construction of a 1 km asphalt pavement surface containing MSWI BAA is calculated as follows: 24 × 0.18 × 1000 = 4320 m^3^. These include 1920 m^3^ of AC-20 asphalt mixture, 1440 m^3^ of AC-16 asphalt mixture, and 960 m^3^ of SMA-13 asphalt mixture. Based on the mix ratio and bulk density of the three types of asphalt mixture shown in Table 6, the amounts of AC-20, AC-16, and SMA-13 asphalt mixture required for a 1 km asphalt pavement surface containing MSWI BAA are 1920 m^3^ × 2.354t/m^3^ = 4519.68t, 3437.28t, and 2321.28t, respectively.

In accordance with the mix ratio of the asphalt mixture containing MSWI BAA, the quantity of each raw material needed for 1 ton of asphalt mixture containing MSWI BAA was calculated, as presented in Table 8.

**Table 8:** The quantity of each material for 1 ton of asphalt mixture containing MSWI BAA.

Type of material	Natural aggregate					A–70#	Mineral powder	BAA
AC–20	Particle size range/mm	0–5	5–10	10–20	–			
	Material quantity/t	0.17	0.28	0.34	–	0.053	0.01	0.2
AC–16	Natural aggregate					A–70#	Mineral powder	BAA
	Particle size range/mm	0–4.75	4.75–9.5	9.5–13.2	13.2–19			
	Material quantity/t	0.17	0.08	0.1	0.37	0.057	0.08	0.2
SMA–13	Natural aggregate					SBS	Mineral powder	BAA
	Particle size range/mm	0–3	3–5	5–10	10–15			
	Material quantity/t	0.01	0.05	0.29	0.45	0.066	0.1	0.1

The raw materials used in the base layer (AC-20) of the 1 km asphalt pavement containing MSWI BAA consists of 3570.55t of aggregate (768.35t of 0—5 mm aggregate, 1265.51t of 5—10 mm aggregate, and 1536.69t of 10—20 mm aggregate), 239.54t of asphalt, 45.2t of mineral powder, and 903.94t of BAA. Similarly, the quantities of raw materials used in the binder and wearing layers of the asphalt pavement can be determined. By summing them, the total quantity of raw materials required for the 1 km asphalt pavement surface layer can be obtained.

#### Ordinary pavement engineering quantity

The total volume of mixture required for constructing the surface layer of a 1 km long ordinary asphalt pavement is the same as that for a 1 km thick asphalt pavement containing MSWI BAA. The use of AC-20, AC-16, and SMA-13 asphalt mixtures for the surface layer of a 1 km thick asphalt pavement is 4717.44 tons, 3585.6 tons, and 2378.88 tons, respectively.

Using the same calculation method as for the asphalt pavement containing MSWI BAA, the quantity of each material in 1 ton of asphalt mixture was calculated, as shown in Table 9.

**Table 9:** The material consumption of 1t asphalt containing MSWI BAA.

Type of mixture	Natural aggregate					A-70#	Mineral powder
AC-20	Particle size range/mm	0–5	5–10	10–20	–		
	Material quantity/t	0.34	0.31	0.33	–	0.039	0.02
AC-16	Natural aggregate					A-70#	Mineral powder
	Particle size range/mm	0–4.75	4.75–9.5	9.5–13.2	13.2–19		
	Material quantity/t	0.35	0.06	0.13	0.37	0.04	0.09
SMA-13	Natural aggregate					A-70#	Mineral powder
	Particle size range/mm	0–3	3–5	5–10	10–15		
	Material quantity/t	0.14	0	0.3	0.45	0.061	0.11

**Fig. 14:**
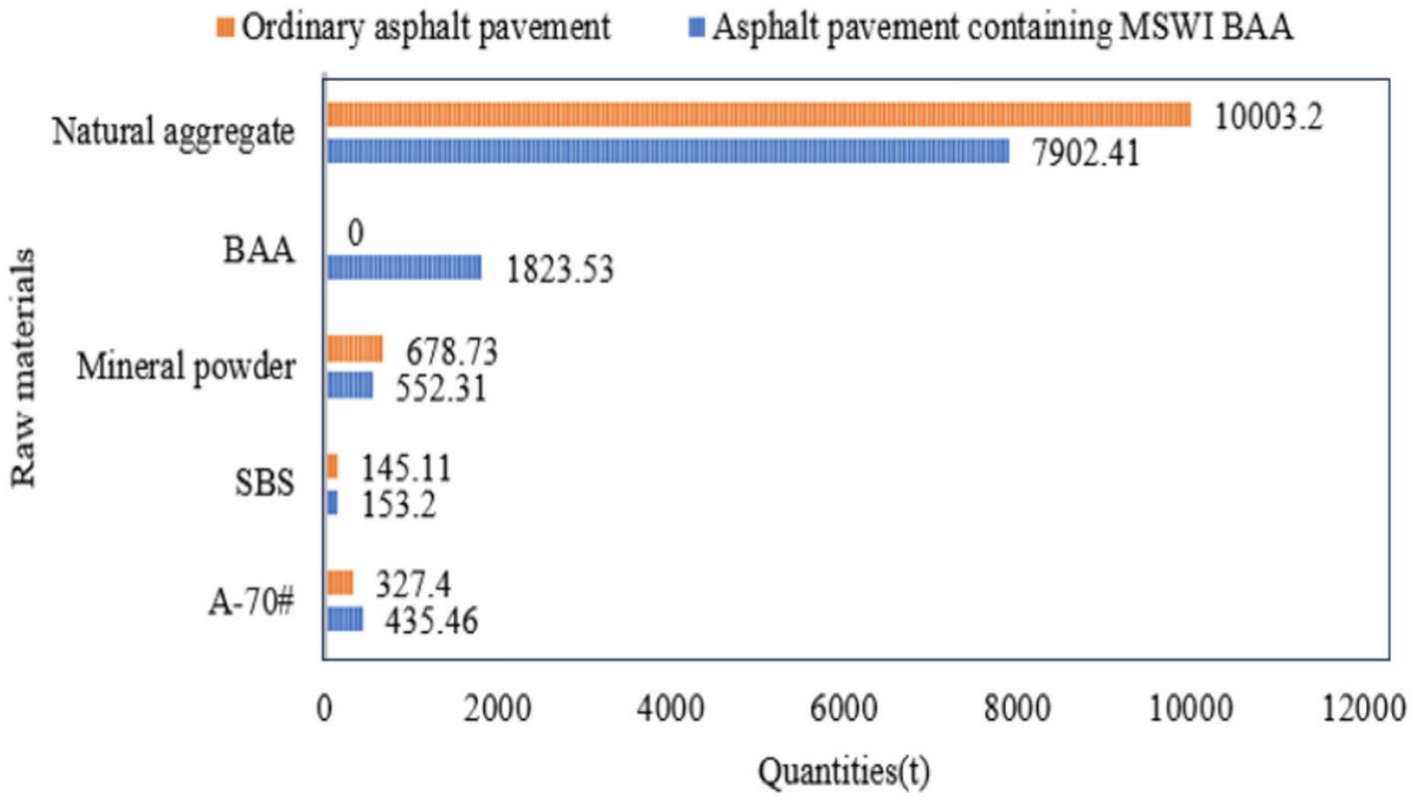
Quantities of raw materials for two types of asphalt pavement. The data in the figure are counted by scientific counting method. The calculated value is based on the original exact data.

By combining the masses of the three asphalt mixtures, the raw material quantities for the surface layer of a 1 km long ordinary asphalt pavement were obtained. A summary and comparison of the raw material quantities for the surface layer of asphalt pavement containing MSWI BAA and the Ordinary asphalt pavement are presented in Fig. 14.

Figure 14 shows that the inclusion of BAA results in an increased asphalt-stone ratio in the asphalt mixture containing MSWI BAA, leading to the use of more asphalt. From an aggregate perspective, replacing natural aggregates with BAA at a mass ratio of approximately 1:1.152 significantly reduces the consumption of natural aggregates. This approach not only minimizes the environmental impact associated with the extraction of natural aggregates but also alleviates the societal burden of managing waste generated from daily life.

## Life cycle inventory calculation for asphalt pavement containing MSWI BAA

### List data calculation of the pavement raw material production stage

#### Quantitative analysis of energy consumption and emissions in the asphalt production stage

**Fig. 15:**
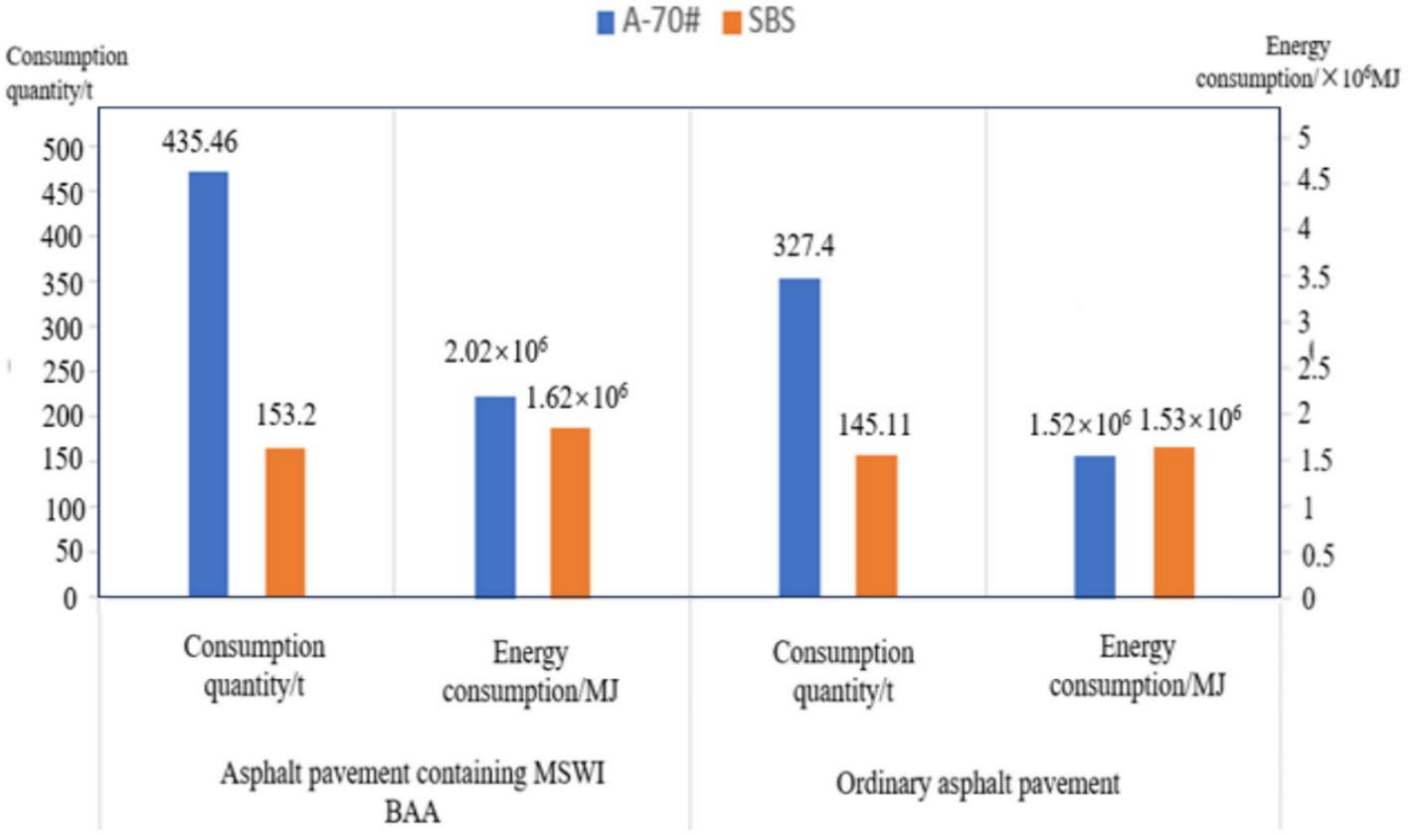
Asphalt production stage energy consumption.

As petroleum asphalt is derived from crude oil distillation, its life cycle is considered to begin from the initial extraction process, according to the European Asphalt Association. The energy consumption and emission factors for petroleum asphalt, including processes such as crude oil extraction, transportation, refining, and storage, are presented in Table 10, along with those for SBS-modified asphalt. Based on the data in the table and using Eq. (2.1) for energy consumption and emissions, the production energy consumption and emissions for both types of asphalt can be determined. The production energy consumption for the two types of asphalt is provided in Fig. 15, and the emissions of gaseous pollutants are shown in Table 11.

**Table 10:** Energy consumption and emission factors of asphalt.

Fuel type	Energy consumption/(MJ•t^-1^)	Emission factor/(g•t^-1^)		
		CO_2_	CH_4_	N_2_O
A-70#	4649.2	4.40 × 10^5^	0.005	0.007
SBS	1.06 × 10^4^	5.52 × 10^5^	1.4	2.1

The above data include the processes of crude oil extraction, transportation, refining, and storage. The data is sourced from *Cleaner production standard Petroleum refinery industry (semi-asphalt flux) (HJ443-2008).*

**Table 11:** Asphalt production emission volume.

Gaseous emission	Emission factor/(g•t^-1^)		Asphalt pavement containing MSWI BAA/g		Ordinary asphalt pavement/g	
	A-70#	SBS	A-70#	SBS	A-70#	SBS
	①	②	③ = ① × 435.46	④ = ② × 153.2	⑤ = ① × 327.4	⑥ = ② × 145.11
CO_2_	4.40 × 10^5^	5.52 × 10^5^	1.91 × 10^8^	8.46 × 10^7^	1.44 × 10^8^	8.01 × 10^7^
CH_4_	0.005	1.4	2.18	214.48	1.637	203.15
N_2_O	0.007	2.1	3.05	321.72	2.29	304.73

#### Quantitative analysis of energy consumption and emissions in the stone production stage

**Fig. 16:**
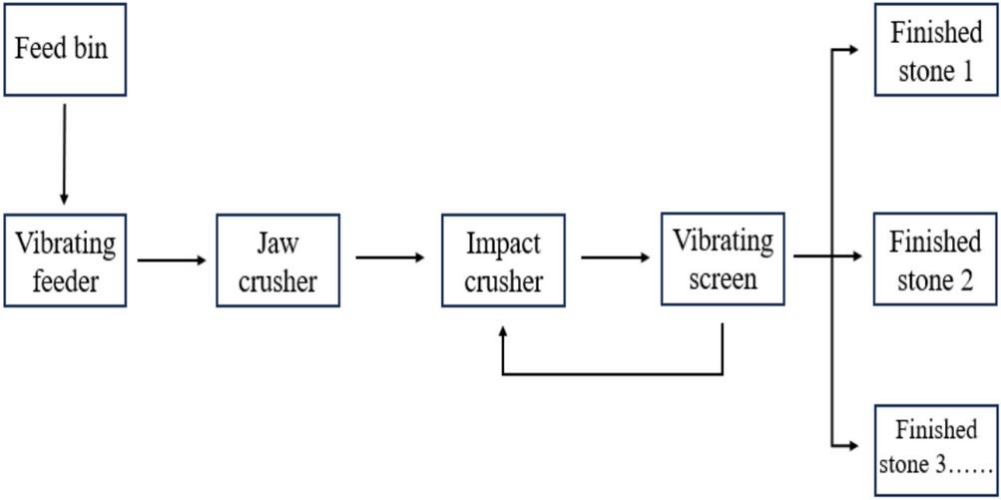
Stone production process flow.

The raw stones are first conveyed to a jaw crusher for coarse crushing via a vibrating feeder. The resulting coarse-crushed stones are then transported to an impact crusher for secondary crushing. Next, the finely crushed stones are conveyed to a vibrating screen for screening to meet various size specifications. Stones that meet the particle size requirements are transported to the finished product stockpile, while those that do not meet the requirements are sent back to the impact crusher for further crushing. This cyclic process is repeated multiple times until all stones meet the particle size requirements. The stone production process is illustrated in Fig. 16.

The primary machinery employed in the production of natural aggregates is typically powered by electricity, making electricity consumption the dominant factor in the overall energy consumption of the aggregate production process. This study examines the key technical specifications of commonly used aggregate production lines in China. By considering their processing capacity and total installed power, the energy consumption per unit output of the production lines under ideal conditions is calculated, as presented in Table 12.

**Table 12:** Unit energy consumption of aggregate production line.

Maximum feed /mm	Process power/(t•h^-1^)	Installed net horsepower power /kw	Energy consumption /(kWh•t^-1^)
340	30	120	4
340	50	140	2.8
420	80	200	2.5
480	100	280	2.8
560	150	300	2
630	200	350	1.75
630	250	400	1.6
630	300	450	1.5
700	350	500	1.43
700	450	600	1.33

**Fig. 17:**
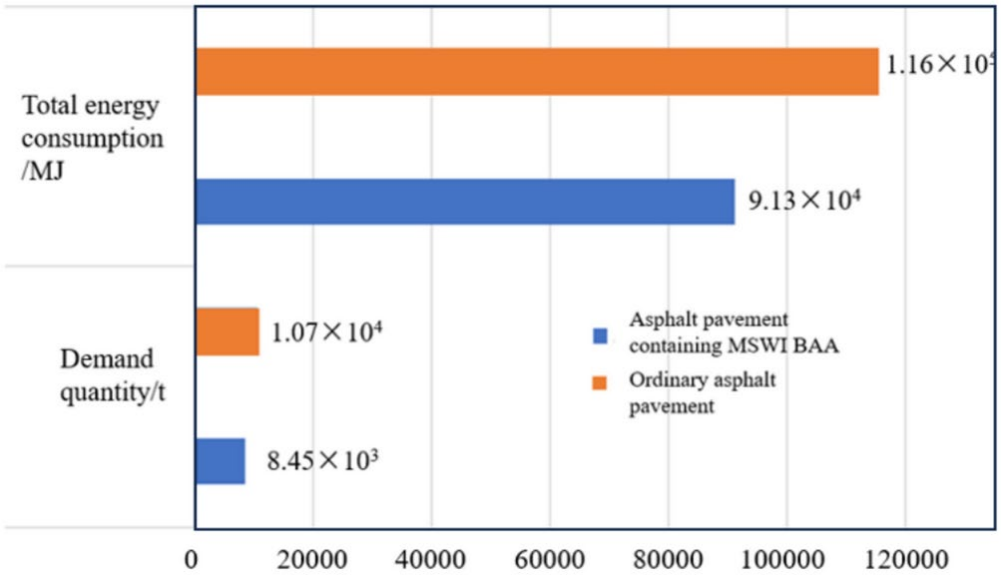
Total energy consumption in the natural aggregate production stage.

According to the data in Table 12, the 75th percentile of energy consumption per unit output for domestic aggregate production lines is 2.8 kWh/t. For convenience in calculation, 3 kWh/t is used as the representative value to calculate the energy consumption in the aggregate production process. The electricity consumption is calculated based on an equivalent heat value of 3.6 MJ per kWh, meaning that under average ideal conditions, the energy consumption in the aggregate production stage is approximately 10.8 MJ/t. The total energy consumption and emissions in the natural aggregate production stage for the two types of asphalt pavement are shown in Fig. 17 and Table 13, respectively.

**Table 13:** Total emissions in the natural aggregate production stage.

Gaseous emission	Emission factor/(g•t^-1^)	Asphalt pavement containing MSWI BAA/g	Ordinary asphalt pavement/g
①	②	③ = ② × 8454.72	④ = ② × 10,681.93
CO_2_	2487	2.1 × 10^7^	2.66 × 10^7^
CH_4_	0.03507	296.51	374.62
N_2_O	0.05076	429.16	542.21

2487 = 829 × 3, 0.0466 = 0.01169 × 3, 0.05076 = 0.01692 × 3.

#### Quantitative analysis of energy consumption and emission in the BAA production stage

##### Asphalt production stage energy consumption calculation

Before utilizing of bottom ash comprehensively, it is necessary to sort it, separate metal components, remove impurities, and obtain aggregates of various particle sizes. The bottom ash sorting process primarily includes dry and wet methods; in this paper, the wet sorting method is generally employed for bottom ash treatment.

The typical scale of bottom ash processing is 300 t/d, equivalent to 12.5 t/h. The total power of the bottom ash sorting process system is 250 kW, and its energy consumption is calculated as E = 250 kW ÷ 12.5t/h = 20 kWh/t.

Based on earlier calculations for the quantity of BAA required for a 1 km long asphalt pavement containing MSWI BAA, which is 1823.53 tons, the energy consumption for electricity, calculated at a 1 kWh/t equivalent heat value of 3.6 MJ, is as follows:

$$1823.53t \times 20kWh/t \times 3.6MJ = 1.31 \times 10^{5} MJ$$

##### Asphalt production stage emission calculation

The BAA production stage includes the processing and screening of bottom ash. The machinery used in this process primarily consumes electricity.

The environmental emissions associated with this stage are detailed in Table 14.

**Table 14:** Environmental emissions from bottom ash processing and screening.

Gaseous emission	Emission factor/(g·t^-1^)	Asphalt pavement containing MSWI BAA/g
①	②	③ = ② × 1823.53
CO_2_	16,580	3.02 × 10^7^
CH_4_	0.2338	426.34
N_2_O	0.3384	617.08

16,580 = 829 × 20, 0.2338 = 0.01169 × 20, 0.3384 = 0.01692 × 20.

### Calculation and analysis of inventory data in the pavement construction stage

In the current scenario of mechanized construction, energy consumption during the construction process of asphalt pavement primarily comes from the liquid fuel used by various construction machinery and equipment. Correspondingly, environmental emissions mainly arise from associated fuel consumption.

#### Analysis of energy consumption and emission from asphalt mixture mixing

##### Asphalt mixture production energy consumption calculation

The energy consumption during the asphalt mixture production process mainly includes liquid fuel consumption and electricity consumption. According to the quota method of energy consumption calculation, the energy consumption of the asphalt mixture production process is calculated.

AC-20 and AC-16 are medium-grade asphalt mixtures. Based on the specified number of shifts and the specified liquid fuel and electricity consumption per shift, the fuel oil and electricity consumption for the production process of medium-grade asphalt mixture pavement is calculated.

According to the *Highway Engineering Budget Quota (JTG/T3832-2018)*, this study used asphalt mixing equipment with a production capacity of up to 120 t/h. 1000 m^3^ of medium-graded asphalt mixture pavement requires 3.74 shifts. Each shift consumes 3590.4 kg of fuel oil and 1859.23 kWh of electricity. Based on the actual quantity of pavement and considering the heating values of different fuels, the total energy consumption during the production process of a medium-grade asphalt mixture on actual pavement is calculated. Refer to Table 15.

**Table 15:** AC mixture energy consumption.

Production capacity/(t•h^-1^)			Within 120
Shift		①	3.74
Heavy oil	kg/Shift	②	3590.4
Electricity	(kWh)/Shift	③	1859.23
Engineering quantity/m^3^	AC-20	④	1920
	AC-16	⑤	1440
AC-20	Heavy oil/kg	⑥ = ① × ② × ④/1000	2.58 × 10^4^
	Electricity/(kWh)	⑦ = ① × ③ × ④/1000	1.34 × 10^4^
AC-16	Heavy oil/kg	⑧ = ① × ② × ⑤/1000	1.93 × 10^4^
	Electricity/(kWh)	⑨ = ① × ③ × ⑤/1000	1.00 × 10^4^
Energy consumption/MJ	Heavy oil	⑩ = (⑥ + ⑧) × 40.4	1.82 × 10^6^
	Electricity	⑪ = (⑦ + ⑨) × 3.6	8.41 × 10^4^

The energy consumption of SMA-13 mixture was calculated using the mentioned method above. Refer to Table 16 for further details.

**Table 16:** Production energy consumption of SMA asphalt mixture pavement entity.

Asphalt mixing equipment production capacity/(t•h^-1^)			Within 120
Shift		①	4.65
Heavy oil	kg/Shift	②	3590.4
Electricity	(kWh)/Shift	③	1859.23
Cumulative consumption	Heavy oil/kg	④ = ① × ②	1.67 × 10^4^
	electricity/(kWh)	⑤ = ① × ③	8645.42
Sub-item energy consumption	Heavy oil/MJ	⑥ = ④ × 40.4	6.74 × 10^5^
	Electricity/MJ	⑦ = ⑤ × 3.6	3.11 × 10^4^
Energy consumption/MJ		⑧ = (⑥ + ⑦) × 960/1000	6.77 × 10^5^

##### Calculation of environmental emissions during asphalt mixture production

In this paper, continuous drum-type mixing equipment is selected for the asphalt mixture mixing process. The actual quantity of asphalt mixture required for pavement entities, as per Eq. (2.1), is used to calculate the gaseous pollutant emissions during the asphalt mixture production process. Refer to Table 17 for further details.

**Table 17:** Gaseous pollutant emissions in the production process of asphalt mixture.

Pollutant emissions	Emission factor/(kg•t^-1^)	Ordinary asphalt pavement				Asphalt pavement containing MSWI BAA			
		AC-20	AC-16	SMA-13	Total/kg	AC-20	AC-16	SMA-13	Total/kg
	①	② = ① × 4717.44	③ = ① × 3585.6	④ = ① × 2378.88	⑤ = ① + ② + ③ + ④	⑥ = ① × 4519.68	⑦ = ① × 3437.28	⑧ = ① × 2321.28	⑨ = ⑥ + ⑦ + ⑧
CO	0.065	306.63	233.06	154.63	694.32	293.78	223.42	150.88	668.09
CO_2_	16.5	7.78 × 10^4^	5.91 × 10^4^	3.94 × 10^4^	1.76 × 10^5^	7.46 × 10^4^	5.67 × 10^4^	3.83 × 10^4^	1.70 × 10^5^
NO_X_	0.0275	129.73	98.6	65.42	293.75	124.29	94.53	63.84	282.65
SO_2_	0.029	136.81	103.98	68.99	309.78	131.07	99.68	67.32	298.07
CH_4_	0.006	28.3	21.51	14.27	64.09	27.12	20.62	13.93	61.67
VOC	0.016	75.48	57.37	38.06	170.91	72.31	55	37.14	164.45

The emission factors are from US EPA.

#### Asphalt pavement construction machinery and equipment

The machinery used in asphalt pavement construction can be classified into three categories: transport machinery, paving machinery, and compaction machinery. To minimize the need for vehicle changes and unloading in front of the paving machine, as well as to improve the efficiency of asphalt mixture transportation, self-discharging trucks with a load capacity of 15 tons are utilized as transport machinery. The average transport distance is approximately 10 km. According to *the quota of highway engineering machinery shift costs (JTG/T3833-2018)*, for paving machinery, the production capacity of the mixing equipment is within 120 t/h, and the paving width of equipment is within 6.0 m.

#### Energy consumption and emissions of asphalt pavement construction process

##### Calculation of the energy consumption of asphalt pavement construction process

The primary energy consumption in asphalt pavement construction arises from the fuel consumption of machinery, including transport, paving, and compaction equipment. Calculations are based on equipment usage, budget quotas, unit cost quotas, and the net calorific value of fuel (Table 2). Refer to Tables 18, 19 for detailed calculations.

**Table 18:** Asphalt pavement construction energy consumption.

Fuel type	Consumption/kg	Combustion factor	Energy consumption/MJ	Total/MJ
	①	②	③ = ① × ②	
Diesel	1.23 × 10^4^	43.0	5.27 × 10^5^	5.59 × 10^5^
Gasoline	718.37	44.3	3.18 × 10^4^

**Table 19:** Asphalt pavement construction machinery fuel quantity.

Construction process	Construction machinery	AC-16 AC-20	SMA-13	Diesel	Gasoline	AC-16 AC-20		SMA-13	
		Shift/1000m^3^		kg/Shift		Diesel/kg	Gasoline/kg	Diesel/kg	Gasoline/kg
		①	②	③	④	⑤≡① × ③ × 3360/1000	⑥≡① × ④ × 3360/1000	⑦≡② × ③ × 960/1000	⑧≡② × ④ × 960/1000
Asphalt Mixing process	Tire loader within 2m^3^	7.02	8.72	92.86	–	2190.31	–	777.35	–
	Dump truck within 5t	3.89	4.36	–	41.63	–	544.12	–	174.25
Transportation process	15t Dump truck	19.69	19.69	67.89	–	4491.49	–	1283.28	–
Paving process	6.0 m Asphalt mixture paver	3.96	5.01	46.63	–	620.44	–	224.27	–
Compaction process	6–8t Light wheel roller	7.79	9.85	19.33	–	505.951	–	182.78	–
	12–15t Light wheel roller	5.84	9.85	40.46	–	793.922	–	382.59	–
	9–16t Tire roller	3.8	–	33.71	–	430.409	–	–	–
	Vibratory roller within 15t	–	4.89	80.92	–	–	–	379.87	–
	Total	–	–	–	–	9032.52	544.12	3230.15	174.25

##### Calculation of emissions in asphalt pavement construction

Emissions in asphalt pavement construction primarily consist of emissions from machinery fuel consumption and fugitive emissions during the transport and construction of asphalt mixtures. This study focuses on calculating emissions from the combustion of machinery fuel during asphalt pavement construction.

Diesel engines are commonly used in asphalt pavement construction machinery, resulting in the emission of various gaseous pollutants and particulate matter from diesel fuel combustion. Table 19 shows the fuel consumption of asphalt pavement units, and the fuel emission factors are utilized based on the net calorific value provided in Table 2 to calculate the emissions from machinery fuel combustion during the construction process. Refer to Table 20 for more detailed information.

**Table 20:** Construction machinery fuel combustion emissions.

Gaseous emission	Emission factor/(g•kg^-1^)		Emission quantity/g		Total/g
	Diesel	Gasoline	Diesel	Gasoline	
	①	②	③ = ① × 12,262.7	④ = ② × 718.37	
CO_2_	3160	2990	3.88 × 10^7^	2.15 × 10^6^	4.1 × 10^7^
CH_4_	0.128	0.129	1569.63	92.67	1662.3
N_2_O	0.026	0.026	318.83	18.68	337.51

### Calculation and analysis of road operation maintenance stage list data

In the operational stage, roads are susceptible to two major types of damage: rutting and cracking. Rutting refers to the permanent deformation of the road surface in the wheel path of the traffic lane. On highways and primary roads with asphalt surfaces, rutting primarily occurs in the asphalt layer and accounts for approximately 90% of total deformation. Compared to other types of damage, rutting significantly impacts on the quality and lifespan of asphalt road surfaces, directly affecting traffic safety. Therefore, this study focused on analyzing rutting damage in asphalt road surfaces containing BAA.

#### Rutting evaluation methods and standards

Currently, in China, the condition of asphalt pavements is evaluated using rutting depth. For high-grade highway asphalt pavements, the allowable rutting depth typically ranges from 10 to 15 mm.

4.1 $$RDI = \left\{ \begin{gathered} \, 100 - \, \alpha_{0} RD\,\,\,\,\,\,\,\,\,\,\,,\,\,\,\,\,\,\,\,\,RD \le RD_{\alpha } \hfill \\ 60 - \alpha_{1} \,RD - RD_{\alpha } )\,\,\,\,\,\,,\,\,\,\,\,\,\,\,\,\,\,RD_{\alpha } \le RD \le RD_{\beta } \hfill \\ \, 0RD\,\,\,\,\,\,\,\,\,\,\,\,\,,\,\,\,\,\,\,\,\,\,\,RD_{\beta } \hfill \\ \end{gathered} \right.$$

where, *RD *- Rutting Depth, mm; *RD*_*α*_ - Rutting depth parameter, commonly using 20 mm; α_0_—model parameter, commonly using2.0; α_1_—model parameter, commonly using4.0.

The rutting depth index (RDI) is used to evaluate asphalt rutting calculated according to Eq. (4.1). The corresponding evaluation standards are provided in Table 21.

**Table 21:** Current specifications for evaluating road rutting depth.

	Assessment grade				
	Excellent	Good	Fair	Poor	Fail
Rutting depth index RDI	≥ 90	≥ 80	≥ 70	≥ 60	< 60
		< 90	< 80	< 70	
Rutting depth RD/mm	≤ 5	> 5	> 10	> 15	> 20
		≤ 10	≤ 15	≤ 20	

#### Energy consumption calculation based on the rutting damage

To simplify the analysis process and avoid uncertainties caused by materials and construction processes in rutting maintenance analysis, this study assumes that rutting damage does not exceed the thickness of the asphalt pavement surface layer. Two types of asphalt pavement surface layers are considered: the SMA-13 asphalt mixture. By conducting rutting tests, the average dynamic stability of the SMA-13 asphalt mixture with different proportions of BAA was determined.

**Fig. 18:**
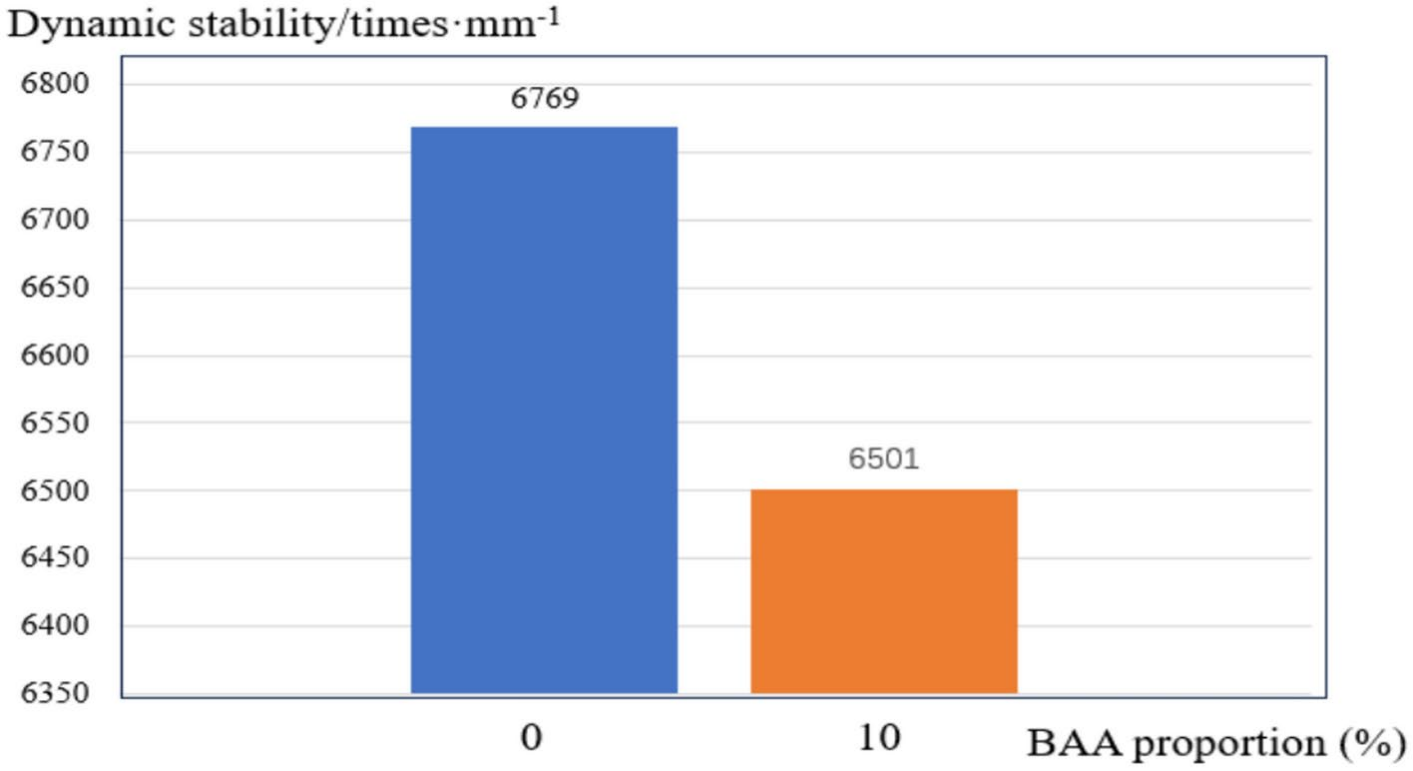
Dynamic stability under different BAA proportions.

The dynamic stability of the asphalt mixture containing MSWI BAA decreased by approximately 3.96% compared to that of the regular asphalt mixture, as shown in Fig. 18.

Assuming a design service life of 15 years for asphalt pavement with maintenance every 5 years, the following simplifications are adopted for calculations:

1. The pavement material is uniformly damaged at any location simultaneously.
2. The rutting damage criterion is set at 20 mm, and the maintenance procedure involves milling and overlaying with a thickness of 40 mm. Milling is performed on a single lane width of 3.75 m for a double lane with a total road width of 15 m.
3. The milled and overlay material is identical to the original surface layer material.

Using the calculation method for asphalt pavement quantity, the volume of asphalt pavement milled is found to be 600 m^3^.

The materials used for milling and overlaying are listed in Table 22. The energy consumption for a single maintenance operation of the asphalt pavement is shown in Table 23, where 1 represents asphalt pavement containing MSWI BAA and 2 represents ordinary asphalt pavement.

**Table 22:** Single maintenance material dosage of asphalt pavement.

Material type		SBS	Natural aggregate	BAA	Miner powder	Total
SMA-13 asphalt mixture consumption/t	Asphalt pavement containing MSWI BAA	95.75	1160.64	145.1	145.1	1450.8
	Ordinary asphalt pavement	90.70	1323.25	0	163.55	1486.8

**Table 23:** Single maintenance material energy consumption of asphalt pavement.

Raw materials	Consumption amount/t		Unit energy consumption/(MJ•t^-1^)	Energy consumption/MJ	
	1	2		1	2
	①	②	③	④ = ① × ③	⑤ = ② × ③
SBS	95.75	90.70	1.06 × 10^4^	1.01 × 10^6^	9.59 × 10^5^
Natural aggregate (miner powder inclusion)	1305.74	1486.8	10.8	1.41 × 10^4^	1.61 × 10^4^
BAA	145.1	0	72	1.04 × 10^4^	0
Total				11.04 × 10^6^	9.75 × 10^5^

Tables 24, 25 illustrate the total number of shifts used in a single paving operation and the total consumption and mechanical energy consumption, respectively, based on the calculation method of energy consumption during the asphalt pavement construction stage.

**Table 24:** Asphalt pavement single maintenance machinery shift number and consumption.

Machinery	Shift	Heavy oil/kg	Electricity/(kWh)	Diesel/kg	Gasoline/kg
	①	②	③	④	⑤
Asphalt mixing equipment within 120	2.79	2154.24	1115.54	–	–
Tire loader within 2m^3^	5.232	–	–	55.72	–
Dump truck within 5 t	2.616	–	–	–	24.98
15t Dump truck	11.814	–	–	40.73	–
6.0 m Asphalt paver	3.006	–	–	27.98	–
6–8 Ton vibratory roller	5.91	–	–	11.60	–
12–15 Ton vibratory roller	4.434	–	–	24.28	–
Vibratory roller within 15t	2.934	–	–	48.55	–
Total		2154.24	1115.54	229.08	24.98

**Table 25:** Single maintenance machinery energy consumption of asphalt pavement.

Fuel	Consumption	Combustion factor	Energy consumption/MJ	Total/MJ
Heavy oil/kg	2154.24	40.4	8.70 × 10^4^	1.02 × 10^5^
Electricity/(kWh)	1115.538	3.6	4015.94	
Diesel/kg	229.08	43.0	9850.44	
Gasoline/kg	24.978	44.3	1106.53	

#### Environmental emission calculation based on rutting damage maintenance

##### Calculation of the environmental emission of the raw materials

The environmental emissions for the maintenance of rutting damage were calculated using an SMA-13 asphalt mixture, with total quantities of 2901.6 t for asphalt pavement containing MSWI BAA and 2973.6 t for ordinary asphalt pavement; the results are presented in Tables 26, 27, 28 present.

**Table 26:** Emissions from rutting maintenance of two types of asphalt pavements using SBS.

Gaseous emission	Emission factor/(g•t^-1^)	Asphalt pavement containing MSWI BAA/g	Ordinary asphalt pavement/g
	①	② = ① × 95.75 × 2	③ = ① × 90.695 × 2
CO_2_	5.52 × 10^5^	1.06 × 10^8^	1.00 × 10^8^
CH_4_	1.4	268.1	253.95
N_2_O	2.1	402.15	380.92

**Table 27:** Natural aggregate emissions in rutting maintenance stage of asphalt pavement.

Gaseous emission	Emission factor /(g•t^-1^)	Asphalt pavement containing MSWI BAA/g	Ordinary asphalt pavement/g
①	②	③ = ② × 1305.74 × 2	④ = ② × 1486.8 × 2
CO_2_	2487	6.49 × 10^6^	7.4 × 10^6^
CH_4_	0.03507	91.58	104.28
N_2_O	0.05076	132.56	150.94

**Table 28:** Emissions during maintenance of rutting in asphalt pavement containing MSWI BAA.

Gaseous emission	Emission factor/(g•t^-1^)	Bottom ash asphalt/kg
①	②	③ = ② × 145.1 × 2
CO_2_	16,580	4811.52
CH_4_	0.2338	0.07
N_2_O	0.3384	0.1

##### Milling construction environmental emission

A continuous drum-type mixing device is selected for maintenance and overlaying in response to rutting damage. Based on the quantity of asphalt mixture used for the pavement overlay and Eq. (2.1), the emission of gaseous pollutants can be calculated, as shown in Table 29.

**Table 29:** Gas emission during the asphalt mixture maintenance and overlaying process.

Pollutant emission	Emission factor/(kg•t^-1^)	Asphalt pavement containing MSWI BAA/kg	Ordinary asphalt pavement/kg
	①	② = ① × 1450.8 × 2	③ = ① × 1486.8 × 2
CO	0.065	188.6	193.28
CO_2_	16.5	4.79 × 10^4^	4.91 × 10^4^
NO_X_	0.0275	79.79	81.77
SO_2_	0.029	84.15	86.23
CH_4_	0.006	17.41	17.84
VOC	0.016	46.43	47.58

Table 29 presents the results for gas emission during the asphalt mixture production process.

**Fig. 19:**
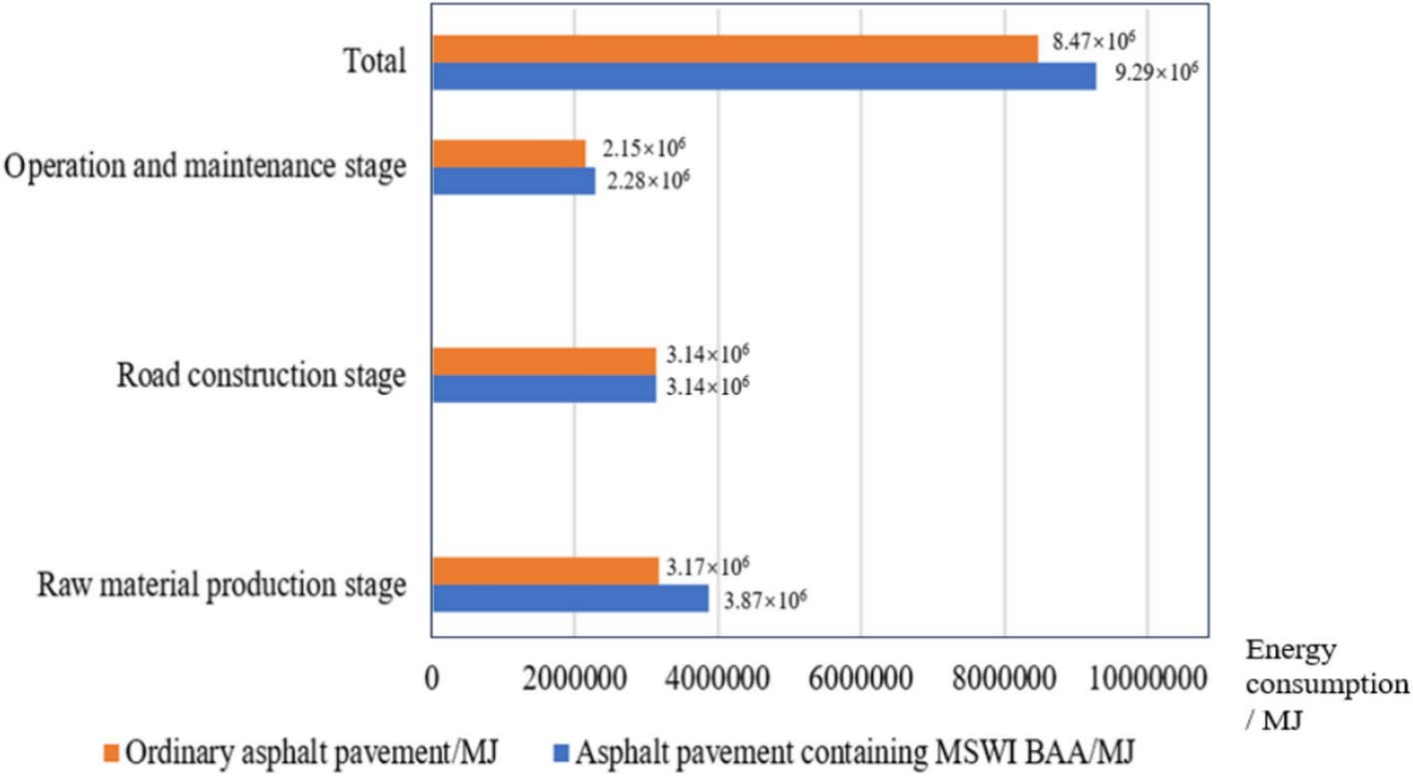
Summary of energy consumption throughout the asphalt life cycle.

According to the road construction process, it is considered that the pavement energy consumption and environmental emissions will not change greatly due to the incorporation of BAA in the demolition stage of pavement structure. Therefore, the study focused on three stages: raw material production, road construction, and operation and maintenance. The summarized results can be found in Table 30 and Fig. 19.

**Table 30:** Summary table of life cycle environmental emissions for asphalt pavement.

Environmental emission	Raw material production stage/g		Road construction stage/g		Operation and maintenance stage/g		Total/g	
	1	2	1	2	1	2	1	2
	①	②	③	④	⑤	⑥	⑦ = ① + ③ + ⑤	⑧ = ② + ④ + ⑥
CO_2_	3.27 × 10^8^	2.51 × 10^8^	2.11 × 10^8^	2.17 × 10^8^	1.6 × 10^8^	1.57 × 10^8^	6.98 × 10^8^	6.25 × 10^8^
CH_4_	939.51	579.41	6.33 × 10^4^	6.58 × 10^4^	1.78 × 10^4^	1.82 × 10^4^	8.2 × 10^4^	8.46 × 10^4^
N_2_O	1371.01	849.23	337.51	337.51	634.71	531.86	2343.23	1718.6
SO_2_	–	–	2.98 × 10^5^	3.10 × 10^5^	8.41 × 10^4^	8.62 × 10^4^	3.82 × 10^5^	3.96 × 10^5^
NO_X_	–	–	2.83 × 10^5^	2.94 × 10^5^	7.98 × 10^4^	8.18 × 10^4^	3.62 × 10^5^	3.76 × 10^5^
VOC	–	–	1.64 × 10^5^	1.71 × 10^5^	4.64 × 10^4^	4.76 × 10^4^	2.11 × 10^5^	2.18 × 10^5^
CO	–	–	6.68 × 10^5^	6.94 × 10^5^	1.89 × 10^5^	1.93 × 10^5^	8.57 × 10^5^	8.88 × 10^5^

“1” represents asphalt pavement containing MSWI BAA; “2” represents ordinary asphalt pavement.

## Life cycle inventory analysis results for asphalt pavement containing MSWI BAA

### Categorization and characterization of environmental impact factors

Based on the types of environmental gas emissions mentioned earlier, they can be classified into three types: greenhouse effect, acidification effect, and harmful gases. Using Eq. (2.2) and the characterization factors in Table 3, the emissions of various gas types in Table 30 were characterized, and the results are shown in Table 31.

**Table 31:** Life cycle environmental emission characterization of asphalt pavement.

Environmental emission types		Greenhouse gases			Total/g	Acidifying gases		Total/g	Harmful gases		Total/g
		CO_2_	CH_4_	N_2_O		SO_2_	NO_X_		VOC	CO	
Characteristic factor		1	25	298		1	0.7		0.64	2.4	
Equivalent factor		CO_2_				SO_2_			1,4-Dichlorobenzene		
Raw material production stage/kg	1	3.27 × 10^8^	939.51	1371.01	3.27 × 10^8^	–	–	–	–	–	–
	2	2.51 × 10^8^	579.41	849.23	2.51 × 10^8^	–	–	–	–	–	–
Road construction stage/kg	1	2.11 × 10^8^	6.33 × 10^4^	337.51	2.13 × 10^8^	2.98 × 10^5^	2.83 × 10^5^	4.96 × 10^5^	1.64 × 10^5^	6.68 × 10^5^	1.71 × 10^6^
	2	2.17 × 10^8^	6.58 × 10^4^	337.51	2.19 × 10^8^	3.10 × 10^5^	2.94 × 10^5^	5.15 × 10^5^	1.71 × 10^5^	6.94 × 10^5^	1.78 × 10^6^
Operation and maintenance stage/kg	1	1.6 × 10^8^	1.78 × 10^4^	634.71	1.61 × 10^8^	8.42 × 10^4^	7.98 × 10^4^	1.40 × 10^5^	4.64 × 10^4^	1.89 × 10^5^	4.82 × 10^5^
	2	1.57 × 10^8^	1.82 × 10^4^	531.86	1.59 × 10^8^	8.62 × 10^4^	8.18 × 10^4^	1.43 × 10^5^	4.76 × 10^4^	1.93 × 10^5^	4.94 × 10^5^
Total/kg	1	6.98 × 10^8^	8.2 × 10^4^	2343.23	7.01 × 10^8^	3.82 × 10^5^	3.62 × 10^5^	6.36 × 10^5^	2.11 × 10^5^	8.57 × 10^5^	2.19 × 10^6^
	2	6.25 × 10^8^	8.46 × 10^4^	1718.6	6.29 × 10^8^	3.96 × 10^5^	3.76 × 10^5^	6.59 × 10^5^	2.18 × 10^5^	8.88 × 10^5^	2.27 × 10^6^

“1” represents asphalt pavement containing MSWI BAA; “2” represents ordinary asphalt pavement.

### Interpretation of the inventory analysis results

#### Energy consumption inventory results

The energy consumption inventory is analyzed stage by stage, considering the calculations for energy consumption in raw material production, road construction, and operation and maintenance.

##### Raw material production stage

The energy consumption summary for each material in the raw material production stage is shown in Table 32.

**Table 32:** Energy consumption of each material in raw material production stage.

Type of pavement		Asphalt pavement containing MSWI BAA/MJ	Ordinary asphalt pavement/MJ
Raw materials	A-70#	2.02 × 10^6^	1.52 × 10^6^
	SBS	1.62 × 10^6^	1.53 × 10^6^
	Natural aggregates	9.13 × 10^4^	1.15 × 10^5^
	BAA	1.31 × 10^5^	0
Total		3.87 × 10^6^	3.17 × 10^6^

**Fig. 20:**
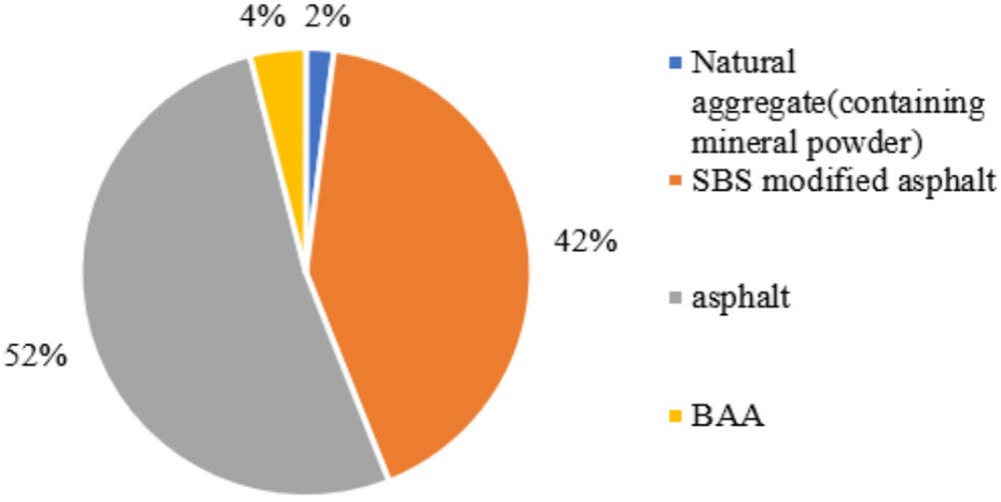
Composition of raw material energy consumption of asphalt pavement containing MSWI BAA.

**Fig. 21:**
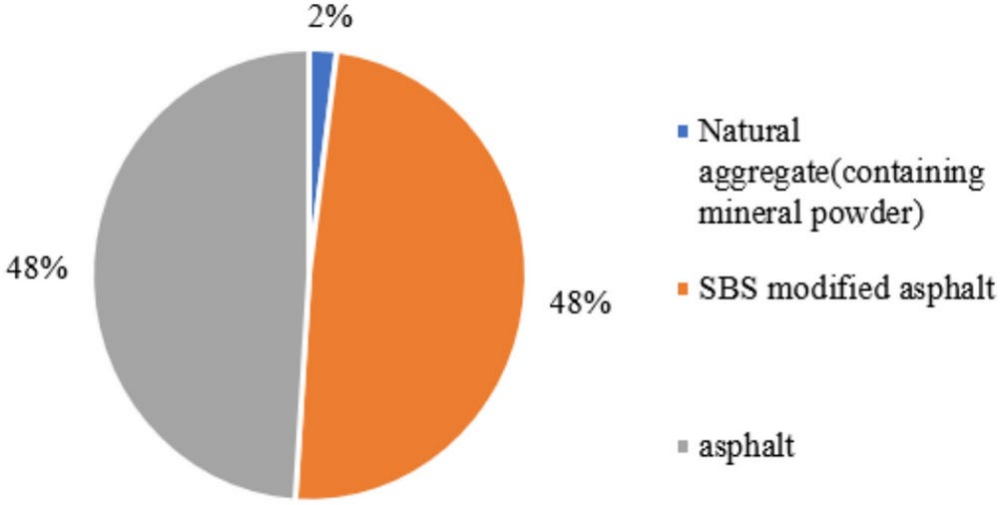
Composition of the raw material energy consumption of ordinary asphalt pavement.

Based on Table 32, Figs. 20 and 21 present the obtained data. The figures clearly indicate that among all the raw materials for the two types of asphalt pavement, the production energy consumption of A-70# is the highest, accounting for 52% and 48% of the raw material energy consumption for asphalt pavement containing MSWI BAA and ordinary asphalt pavement, respectively. The production energy consumption of SBS-modified asphalt accounts for 42% and 48% of the raw material energy consumption for asphalt pavement containing MSWI BAA and ordinary asphalt pavement, respectively. By referring to the raw material energy consumption values in Table 32, it can be inferred that, due to the addition of BAA, the energy consumption of A-70# in asphalt pavement containing MSWI BAA increases by 33%, the energy consumption of SBS-modified asphalt increases by 5.58%, and the energy consumption of natural aggregates decreases by 20.85%, resulting in an overall increase in energy consumption of 21.92%. From the perspective of raw material usage, the addition of BAA significantly influences the consumption of asphalt and natural aggregates. As presented in Fig. 15, Table 32, Figs. 17 and 14, for a 1 km section, compared to Ordinary asphalt pavement, asphalt pavement containing MSWI BAA has an increase of 108.06 tons in A-70# usage, an increase of 8.09 tons in SBS-modified asphalt usage, a decrease of 2100.8 tons in natural aggregate usage, and an additional usage of 1823.53 tons of BAA.

##### Road construction stage

From the energy consumption calculations for the road construction stage, it is evident that the energy consumption in this process is influenced by both the type of asphalt mixture and the fuel type of the construction machinery. Since both asphalt pavement containing MSWI BAA and ordinary asphalt pavement require the same volume of AC-20, AC-16, and SMA-13 asphalt mixture and because the mixing equipment has a production capacity within the range of 120 t/h, the total energy consumption during the road construction stage is comparable, with no significant differences.

##### Operation and maintenance stage

According to Fig. 19, the energy consumption in the maintenance stage for the two types of asphalt pavement is 2.28 × 10^6^ MJ for the asphalt pavement containing MSWI BAA and 2.15 × 10^6^ MJ for the ordinary asphalt pavement. A comparison reveals that the energy consumption of asphalt pavement containing MSWI BAA is 5.7% greater than that of ordinary asphalt pavement. The underlying reason is similar to that in the raw material production stage.

#### Results for the environmental emission inventory

**Fig. 22:**
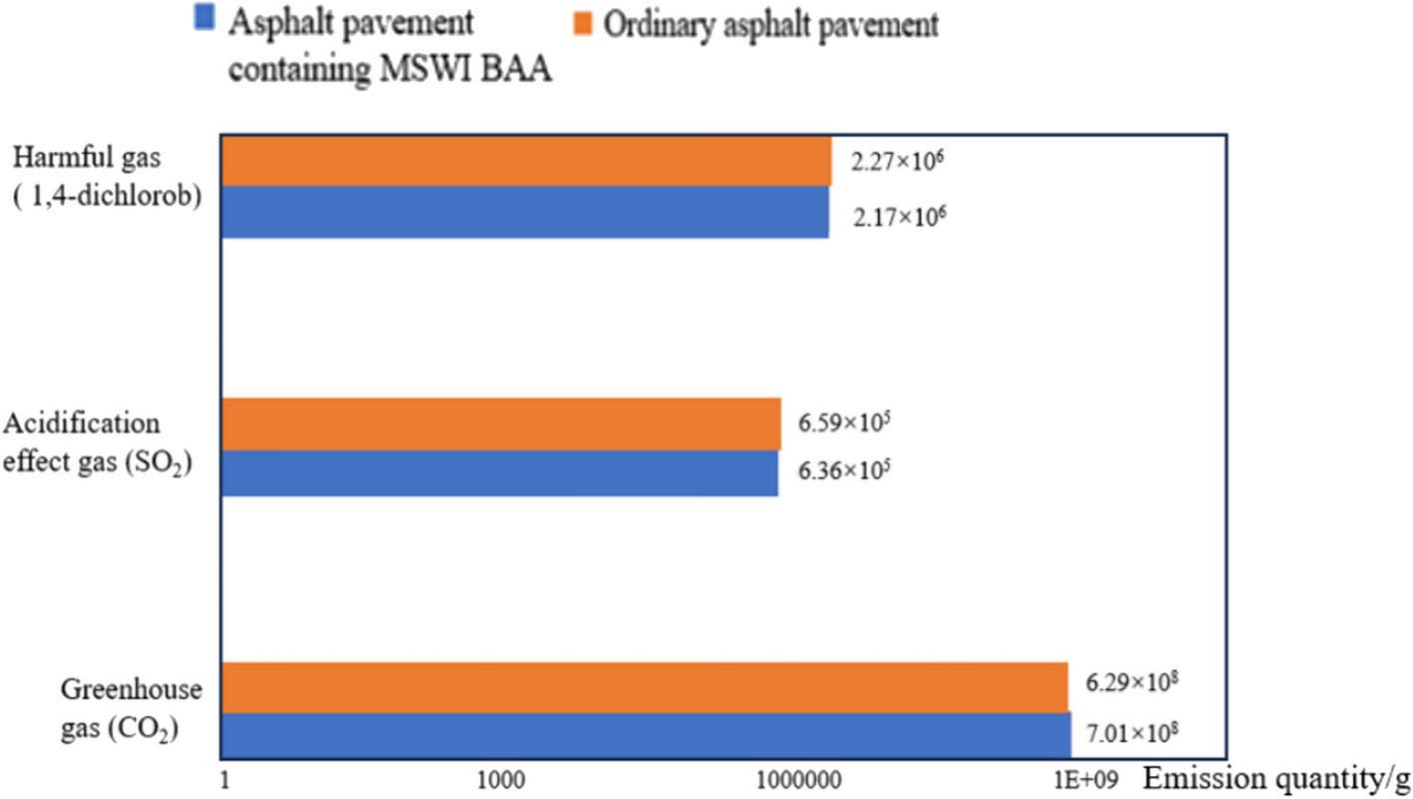
Environmental emissions throughout the life cycle of two asphalt pavements.

According to the environmental emission data provided in Table 31, a comparison of gas emissions between the two characterized asphalt pavements is presented in Tables 33 and Fig. 22. Figure 22 illustrates the overall environmental emissions throughout the entire lifecycle of the two asphalt pavements.

**Table 33:** Asphalt pavement environmental emissions (equivalent).

Raw material production stage		
Gas types	Emission quantity/g	
	Asphalt pavement containing MSWI BAA	Asphalt pavement containing MSWI BAA
Greenhouse gas (CO_2)_	3.27 × 10^8^	2.51 × 10^8^

Construction production stage		
Gas types	Emission quantity/g	
	Asphalt pavement containing MSWI BAA	Asphalt pavement containing MSWI BAA
Greenhouse gas (CO_2)_	2.13 × 10^8^	2.19 × 10^8^
Acidifying gas (SO_2)_	4.96 × 10^5^	5.15 × 10^5^
Harmful gas(1,4-Dichlorobenzene)	1.71 × 10^6^	1.78 × 10^6^

Operation and maintenance stage		
Gas types	Emission quantity/g	
	Asphalt pavement containing MSWI BAA	Asphalt pavement containing MSWI BAA
Greenhouse gas (CO_2)_	1.61 × 10^8^	1.59 × 10^8^
Acidifying gas (SO_2)_	1.40 × 10^5^	1.43 × 10^5^
Harmful gas(1,4-Dichlorobenzene)	4.82 × 10^5^	4.94 × 10^5^

Based on Tables 33, it is evident that, in the raw material production stage, asphalt pavement containing MSWI BAA exhibits higher environmental emissions than ordinary asphalt pavement. This difference is attributed to the inclusion of bottom ash in the raw materials, which leads to increased asphalt material usage and associated emissions from bottom ash processing. However, during the construction and operation maintenance stages, there is minimal disparity in the emissions of the three types of environmental pollutants between the two asphalt pavements.

As shown in Fig. 22, for both asphalt pavement containing MSWI BAA and conventional asphalt pavement, the emissions of the three impact categories follow the order: greenhouse gases > harmful gases > acidification effects. The emissions of greenhouse gases are two orders of magnitude higher than those of harmful gases and three orders of magnitude higher than those of acidification gases. According to Figure 6.3, it can be seen that the emissions of the same type of gas are relatively similar throughout the entire lifecycle of both asphalt pavements.

## Conclusion

In the raw material production stage, the energy consumption of asphalt pavement containing MSWI BAA is 21.92% higher than that of conventional asphalt pavement. The large energy consumption gap in the raw material production stage may be due to the higher oil-stone ratios in the asphalt mixtures containing BAA compared to those with natural aggregates. Specifically, the oil-stone ratios in the AC-20, AC-16, and SMA-13 asphalt mixtures with bottom ash are 36.6, 45.2, and 9.2% higher, respectively, than those in mixtures with natural aggregates.

In the operation and maintenance stage, the energy consumption of asphalt pavement containing MSWI BAA is 5.7% higher than that of conventional asphalt pavement. This study only considers the energy consumption in the maintenance stage, which mainly comes from the production of asphalt mixtures. The absorption effect of the pavement on vehicle exhaust gases is not taken into account. The porous nature of bottom ash aggregate leads to higher asphalt consumption and could absorb more harmful gases.

To address the issues mentioned above, two aspects can be considered:

1. The traditional Marshall design method is still in use, resulting in excessive asphalt content in MSWI BAA asphalt pavements. Could new mix design methods be adopted?
2. According to Mahinsasa’s research, bottom ash can be used as a solid adsorbent for SO_2_. So we should think about what is the actual effect of MSWI BAA asphalt pavements in absorbing vehicle exhaust gases^24^?

Additionally, during the raw material production stage, asphalt pavement containing MSWI BAA uses 2100.8 tons less natural aggregate and consumes an additional 1823.53 tons of bottom ash aggregate. This not only saves natural aggregate resources but also helps address the challenges associated with the disposal of recycled municipal solid waste.

Through this study, we are inspired to consider how to reduce the negative impacts caused by bottom ash while enhancing its positive effects in the research on MSWI BAA asphalt pavements.

## Data Availability

All data generated or analysed during this study are included in this published article (and its Supplementary information files).
